# A six-month prospective, randomised, double-blinded, placebo-controlled, crossover, dietary trial design to investigate the potential of psychobiotics on seizure semiology and comorbidities in canine epilepsy: study protocol

**DOI:** 10.1186/s12917-023-03609-0

**Published:** 2023-03-03

**Authors:** Teresa Schmidt, Sebastian Meller, Nina Meyerhoff, Friederike Twele, Brian Zanghi, Holger Andreas Volk

**Affiliations:** 1 Department of Small Animal Medicine and Surgery, University of Veterinary Medicine Hannover, Hannover, Germany; 2 Centre for Systems Neuroscience, University of Veterinary Medicine Hannover, Hannover, Germany; 3 Research and Development, Nestlé Purina PetCare, St. Louis, MO USA

**Keywords:** Epilepsy, Comorbidities, Anxiety, Quality of life, Microbiome-gut-brain axis, *Bifidobacterium longum*

## Abstract

**Background:**

Epilepsy is the most common chronic neurological disease in dogs. More than two-thirds of these patients suffer from associated behavioural comorbidities. The latter could have their origin in partially overlapping pathomechanisms, with the intestinal microbiome as a potential key link between them. The current arsenal of drugs for epilepsy management remains limited. Most canine patients continue to have seizures despite treatment and the occurrence of comorbidities is not sufficiently addressed, limiting quality of life of affected dogs and owners. Therefore, novel additional epilepsy management options are urgently needed. The microbiome-gut-brain axis may serve as a new target for the development of innovative multimodal therapeutic approaches to overcome current shortcomings in epilepsy management.

**Methods:**

A six-month prospective, randomised, double-blinded, placebo-controlled, crossover, dietary trial was designed to investigate the potential of the psychobiotic *Bifidobacterium longum* on behavioural comorbidities in canine epilepsy. Seizure semiology will be evaluated as a secondary outcome measure. Thirty-four privately owned dogs are planned to be included in the ongoing study meeting the following inclusion criteria: Dogs displaying increased anxiety/fear behaviour since the start of the idiopathic epilepsy. Tier II confidence level of the International Veterinary Epilepsy Task Force for the diagnosis of idiopathic epilepsy, with a maximum seizure interval of 3 month and a minimum of three generalised seizures within that period and chronically treated with at least one antiseizure drug without improvement in seizure frequency Each dog will receive the allocated supplement (probiotic vs. placebo) alongside its normal diet for a 3-month period. After a three-week wash out period, the second phase starts by administering the respective other supplement for another 3 months.

**Discussion:**

The current study considers modern high-quality standards for epilepsy medication trials. Common biasing effects should be limited to a possible minimum (regression-to-the mean effect, placebo effect, observer effect), ensuring a high validity and accuracy of the acquired results, thus enabling a representative nature of the efficacy of *Bifidobacterium longum* as add-on supplement for dogs suffering from epilepsy and its comorbidities. This publication should provide a description of the study procedure and data acquisition methods, including prognosed statistical analysis.

**Supplementary Information:**

The online version contains supplementary material available at 10.1186/s12917-023-03609-0.

## Background

Epilepsy is the most common chronic neurological disease in humans and dogs [1, 2]. Around two out of three affected dogs do not become seizure free with the currently available pharmacological treatment [3]. Poor seizure control has a negative impact on the overall quality of life (QoL) and on life expectancy, and it causes an enormous psychological and physical stress for dogs and owners [4–6]. Neurological disorders have been associated with cognitive and neurobehavioural comorbidities in humans [7, 8]. Therefore it is assumed that partially overlapping pathomechanisms with epilepsy exist [7]. The same neurobehavioural impairments are known in canine epilepsy [9, 10]. Around 71% of dogs, especially drug-resistant canine patients, show additional behavioural changes like an increase in fear and anxiety-like behaviour, aggression controlling, abnormal reactivity, attachment disorder, demented and apathetic behaviour [11]. In contrast to the episodic seizures, the comorbidities remain present during the interictal phase, affecting the QoL of the dogs and their owners [12]. New holistic therapeutic approaches are needed to improve QoL, drug-response and comorbidities.

**Fig. 1:**
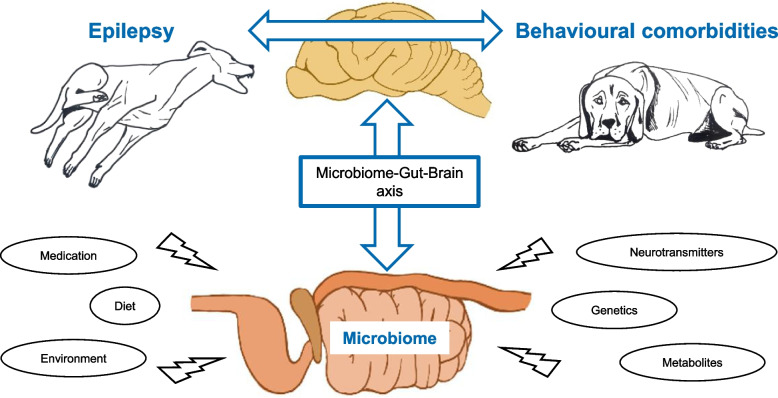
An overview of the microbiome-gut-brain axis and its correlation with epilepsy and associated neurobehavioural comorbidities. Epilepsy and neurobehavioural abnormalities could have their origin in partially overlapping pathomechanisms, with the intestinal microbiome as a potential key link between them. A bidirectional crosstalk between the intestinal flora and the brain is mediated via the microbiome-gut-brain axis. The intestinal microbiome is influenced by multiple internal and external factors (medication, diet, environment, neurotransmitters/metabolites, genetics). It could provide a new complementary target for therapeutic intervention in canine epilepsy and associated behavioural comorbidities

In recent years, there has been an increasing interest in the research field of neurogastroenterology. Evidence from human and animal cases suggests that specific changes in the intestinal flora cause neurodegenerative diseases, modify host behaviour and seizure semiology [13–17]. A bidirectional crosstalk between the intestinal flora and the brain is mediated via the microbiome-gut-brain axis [18] (Fig. 1). The microbiome-gut-brain axis enables the communication through multiple pathways, including the vagus nerve (vagal stimulation), the circulatory system (neurotransmitters, hormones, metabolites, immune signalling) and the immune system (microbial-associated molecular patterns and metabolites) [14, 18].

In simple terms epilepsy is thought to be caused by an imbalance of excitatory and inhibitory neurotransmission [19]. Common pathogenic mechanisms seem to induce neurotransmitter deviations, which evoke not only epileptic seizures, but also psychiatric comorbidities in humans and behavioural changes in canine epilepsy [11, 20]. Previous studies have reported an effect of the intestinal microbiome on the host’s neurotransmitter system [21–23]. For example, serotonin functions as an important neurotransmitter in the microbiome-gut-brain axis, with anticonvulsant properties and a major effect on mood and cognition [21, 24–27]. The host’s serotonin synthesis is influenced by its intestinal flora, which regulates the availability of a key precursor [21]. Furthermore, a microbiome impact on γ-aminobutyric acid (GABA) was shown in multiple studies, and since it is the main inhibitory neurotransmitter of the central nervous system (CNS), the results are promising for the treatment of epilepsy and its comorbidities [22, 23]. Probiotic administration *(Lactobacillus rhamnosus)* modified the central GABA receptor expression and emotional behaviour in mice via the vagus nerve [22]. Another study revealed cultures of human intestinally derived lactic acid bacteria (lacto- and bifidobacteria) metabolised glutamate, the main excitatory neurotransmitter of the CNS into GABA [23].

Many research groups have investigated the correlation between the intestinal microbiome and epilepsy [16, 28, 29]. Medel-Matus et al. [28] found that a varied composition of the intestinal microbiome mediated the stress influence on progression and duration of kindled seizures in rats. Dysbiosis, an alteration in the physiological composition of the intestinal microbiome, is possibly involved in the mechanism of drug-resistant epilepsy [16, 29]. Human patients with drug-resistant epilepsy showed dysbiosis with an abnormally increased abundance of rare flora, while in contrast, the intestinal flora of drug-sensitive patients with epilepsy was similar with that of heathy individuals [16]. In patients with a low seizure frequency, high concentrations of lactobacilli and bifidobacteria were found, which may indicate a protective effect of these bacteria strains [16]. A limited number of recent studies investigated potential microbiome alterations in dogs affected by epilepsy. In one of these studies, drug-naïve epileptic dogs had no deviation in large-scale faecal microbial patterns or difference in the abundance of *Lactobacillus* species in comparison to healthy controls [30]. However, in another study contrary results were revealed. Drug-naïve epileptic dogs showed a significant reduction of GABA and short-chain fatty acids (SCFAs) -producing bacteria compared to controls [31]. Moreover, a decrease in bacteria, which are assumed to mediate a brain protective effect, were detected in affected dogs [31]. Those alterations remained stable during antiseizure drug (ASD) treatment [31]. These findings are consistent with a third study, where solely slight modifications of the microbiome during ASD therapy were identified [32]. Additionally, an increase in faecal SCFAs linked to the treatment response was observed, indicating functional alterations in the microbiome of dogs affected by epilepsy [32]. Another study examined the influence of a dietary intervention on the microbiome of drug-resistant epileptic dogs [33]. A significant modification of the lipidome and microbiome species richness in the treated dogs was revealed, including an increase of a bacterium associated with positive behaviour enhancement [33]. The presented studies suggest, that dysbiosis and therapy-induced alterations in the gastrointestinal microbiome might play an important role in canine epilepsy.

Relating to the partially overlapping pathomechanism of epilepsy and behavioural changes, dysbiosis could be a key link between those co-occurring diseases. Mondo et al. [34] identified dysbiosis in fearful and aggressive dogs and assumed behavioural deviations due to production of neuroactive metabolites of the altered intestinal microbiome. Moreover, a large volume of published studies have demonstrated the impact of the intestinal microbiome on anxiety-like and depression-like behaviour in rodents, focussing on the effect of infection and gut inflammation, the influence of administered probiotics and the absence and reconstitution of intestinal microbiota [35].

Together, these studies outline that a balanced intestinal flora is crucial for normal gut physiology, for proper signalling along the microbiome-gut-brain axis and for the health status of the host [36]. Vice versa, dysbiosis can negatively influence gut physiology, cause improper microbiome-gut-brain axis signalling, negative consequences for CNS function and disease [36]. The intestinal microbiome is influenced by various internal and external factors (e.g. diet, environment, medication, genetics, neurotransmitters and metabolites) [37]. Therefore, it provides a wide range of options for therapeutic intervention and many modification attempts have already been successful.

Through changes in diet, it is possible to modify the composition and function of the intestinal microbiome and the behaviour of the host indirectly [38, 39]. Administering a special diet in dogs with behavioural abnormalities altered their neuroendocrine serum parameters, including neurotransmitters which are associated with stress and anxiety [38]. The supplementation of ω-3 and ω-6 fatty acids in pigs and rodents caused changes in the serotonergic and dopaminergic system, with a secondary effect on their behaviour and cognition [39]. A direct modification of the microbiome can be achieved by using prebiotics, probiotics, psychobiotics (probiotics that influence host behaviour), synbiotics, faecal microbiota transplantation (FMT) and bacteriotherapy [40–42]. These interventions reduce stress, anxiety/−like and depression/−like behaviour and improve QoL in humans and rodents [14, 22, 43–46]. One example of a psychobiotic is *Bifidobacterium longum* which has been shown to positively impact physiological and behavioural responses to typical environmental stimuli (e.g. unfamiliar people and change in routine) in dogs with anxious behaviours [47]. Six weeks after administration the dogs were calmer (lower heart rate), less reactive (lower salivary cortisol) and had an improved affective state (higher heart rate variance) [47].

Administering medium chain triglyceride (MCT)-enriched diets is an effective – adjunct option in dogs, humans and rodent with drug-resistant epilepsy, due to its inhibition of excitatory neurotransmission [48–51]. An abnormal energy metabolism in epileptogenic brain areas was discovered in some epilepsy types, resulting in an energy deficiency which may contribute to dysfunction in cerebral cell activity contributing to hyperexcitability causing seizures [51]. The anticonvulsant mechanism of MCT-enriched diets underlies their metabolic effect circumventing this deficit and additional antioxidative properties [51]. Furthermore, consumption of MCT-enriched diets in dogs and ketogenic diet in mice alters their microbiome, which subsequently modifies the systemic host metabolism linked with seizure protection [33, 52]. These previous findings suggest a supplementary microbiome-mediated antiepileptic effect of ketogenic diets. The effect was missing in germ-free and antibiotically treated mice and could be transferred to a control diet group either via FMT of ketogenic diet fed to mice or via probiotic supplementation of ketogenic diet enriched bacterial strains [52].

Further evidence indicates that FMT provides therapeutic success in epilepsy treatment [28, 53]. A case report described a patient who had been suffering from seizures for 17 years who had intestinal microbiota transferred via FMT to treat Morbus Crohn’s disease [53]. This patient was subsequently seizure-free during the 20-month follow-up period [53]. Medel-Matus et al. [28] demonstrated the same antiepileptic effect of an FMT in rats. The progression of kindled seizures in rats with previously stress-induced dysbiosis was reduced by FMT containing the microbiome of sham-stressed animals. In contrast, an FMT from stressed rats to sham-stressed animals resulted in a progression and prolonged duration of seizures. A more precise intervention with the supplementation of a probiotic mixture in human medicine reduced the seizure frequency by more than 50% in 28.9% of the participants with drug-resistant epilepsy, and additionally led to a significant improvement in the QoL [54].

In conclusion, the discussed studies provide evidence that the microbiome-gut-brain axis could be an important link between epilepsy and its comorbidities. The hypothesis of the current study is that feed supplementation with the probiotic *Bifidobacterium longum* leads to an improvement in comorbidities in treated, drug-resistant dogs with idiopathic epilepsy (IE) and may have a positive secondary effect on the semiology of their epilepsy.

The current study will focus on the intestinal microbiome as a new complementary target for therapeutic intervention. An improvement in interictal anxiety-like behaviour, as well as in aggression controlling, abnormal reactivity, attachment disorder, dement and apathic behaviour, and a positive impact on seizure semiology is hypothesised. This new management option could provide another tool to improve epilepsy management in dogs with IE.

## Methods and study design

### Study population

Thirty-four [34] privately owned dogs of both sexes kept as pets recruited via social media, website advertising and study flyers, as well as from external small animal practices and the patient population of the Small Animal Clinic of the University of Veterinary Medicine Hannover, Germany (TiHo) are planned to be included in the ongoing study. Owners interested in the study participation with their dogs will be initially informed about trial modalities by the investigator either via phone or in person at the TiHo. A screening system will be used to identify dogs matching criteria for study entry, prior to assessing the dogs at the clinic. Each dog in this cohort will be allocated a unique study case number and the owners will be asked to complete a standardised clinical history questionnaire online, requesting data regarding signalment, diet, training, seizure semiology, behaviour, QoL, diagnostics and treatment of epilepsy (Additional file 1). Following the eligibility assessment, alternative treatment options will be carefully reviewed and discussed with the owners in collaboration with neurologists at the TiHo. During enrolment and the entire study period, participating dogs will be medically attended by a neurologist of the TiHo. Clinical condition, blood parameters and long-term ASD serum concentration of participating dogs will be monitored at each on-site visit to ensure the highest standard of care. Owners and external veterinarians can contact the investigator directly via email at any time or the TiHo via phone, which will be forwarded to the investigator. After recruitment of half of the dogs, an interim-analysis will be performed by an independent person to evaluate any trends, the power of the study and how this could affect animal welfare. Owners with dogs not matching the inclusion criteria, withdrawing during study participation or successfully completing the trial will be offered alternative/ensuing appointments at the TiHo Neurology department.

### Inclusion criteria

Before study enrolment, the owners will receive a detailed information sheet and sign a consent form to participate in the study and to allow the dog to be videorecorded (Additional file 2). The study protocol will be detailly explained to the owners by the investigator, to ensure that they fully understand alternative treatment options and the impact of the trial on optimisation of the ASD dosage. Eligibility criteria of the study are increases in anxiety/fear-like behaviour of the dogs, since the start of IE and other behavioural abnormalities associated with IE (aggression controlling, abnormal reactivity, attachment disorder, demented and apathetic behaviour) [11]. These are initially assessed via the prior screening system, which generates a preliminary descriptive behavioural profile of each dog, based on previously validated owner-completed behavioural questionnaires [55–59] (Additional file 1). Identified behavioural abnormalities will be further investigated in an in-depth behavioural analysis by conducting a behavioural test and gathering additional physiological parameters during the first study visit (Visit 0). Furthermore, the dogs must meet all criteria of Tier II confidence level of the International Veterinary Epilepsy Task Force (IVETF) for the diagnosis of IE to be included in the study [60]. In this study, two adjustments to IVETF criterion have been applied: the age of the dogs at seizure onset has been increased to 8 years and abnormalities in the interictal physical and neurological examination due to adverse effects of ASD treatment have been tolerated. These modifications will be applied to achieve higher numbers of potential study participants in an appropriate period of time. In all cases unremarkable magnetic resonance imaging [MRI] will be required. MRI examination of potential study participants will be performed by specialists of diagnostic imaging prior enrolment and following the veterinary epilepsy-specific MRI protocol recommended by the IVETF [61]. In addition, each patient needs to have a maximal seizure interval of 3 months, with at least three seizures within that period during its current therapeutic treatment, with at least one long-term ASD in a steady state. If ASD serum concentration is in a steady state, but at a subtherapeutic level and the dog continues having seizures, the owner can choose to adjust the dosage of the current ASD and participate potentially at a later stage. If ASD serum concentration is at the top of the therapeutic range and the dog continues having seizures, the owner can choose to either start with a new add-on ASD and potentially enrol at a later stage, or to participate with their dog in the trial. The neurologist will objectively assist the owner during decision making, listing the pros and cons of each option, ensuring the best options are chosen for the individual dog and owner. It is important to consider that two out of three affected dogs continue having seizures with the currently available treatment, highlighting the relevancy of this trial [3]. On an individual basis, this means that drug-resistant patients continue having seizures, despite multiple dose adjustments and introductions of add-on ASDs. Side effects, which are becoming more likely with higher dosage and polytherapy, must be considered for each patient individually, particularly their negative health effects and impact on the QoL of dogs and owners [12]. In case of treatment failure after dose adjustment or add-on therapy, the owners will still have the option to participate in the study with their dogs, provided all inclusion criteria are met. Exclusion criteria for the study are a known cause of epilepsy (structural epilepsy induced by brain neoplasm, brain trauma, meningoencephalitis, degenerative encephalopathies, malformations shown to induce seizures or cerebrovascular diseases), acute or chronic diseases of the gastrointestinal tract, kidney, liver, or heart failure. Dogs receiving drugs affecting the metabolism of ASDs or having a negative effect on the microbiome will be excluded, as well as pregnant or lactating dogs and those in ongoing breeding projects.

### Study design

The present study comprises a 6-month prospective, randomised, double-blinded, placebo-controlled, crossover, dietary trial investigating the influence of *Bifidobacterium longum* primarily on the behavioural profile (fear and anxiety-like behaviour, aggression, exploratory behaviour, excitability and impulsivity, attachment or attention-seeking behaviour, cognitive impairment, stranger-, dog- and owner-directed behaviour) and secondarily on seizure semiology in comparison with a placebo supplement in dogs with drug-resistant IE.

A power analysis was performed utilizing the statistical power analysis program G*Power (latest ver. 3.1.9.7.; Heinrich Heine University Düsseldorf, Düsseldorf, Germany) [62]. An optimal sample size of 34 participants was calculated. The sample size is in line with previous studies, evaluating either the primary outcome behaviour (anxiety/fear) or the secondary outcome seizure frequency of the current study as major outcome variable [47, 50, 63, 64]. A type I error of 0.05 and a type II error of 0.8 were used for power analysis. The exact effect size cannot be predicted precisely. Considering former studies, a medium effect size of 0.4 was used for calculation [47, 50, 63, 64]. However, it is likely that the effect size will be higher (0.5); in this case, a minimum of 21 dogs is needed. It was therefore decided that after 21 dogs have completed the study, an interim analysis will be performed to consolidate the power analysis.

The study will be conducted in consideration of the “Guidelines to Safeguard Good Scientific Practice and Measures to Be Taken in Case of Suspicion of Scientific Misconduct at the University of Veterinary Medicine Hannover” at the Department of Small Animal Medicine and Surgery, University of Veterinary Medicine Hannover, Germany. An animal test certificate was granted by the Lower Saxony State Office for Consumer Protection and Food Safety (LAVES) (approval number 33.8–42,502-05-19A469).

**Fig. 2:**
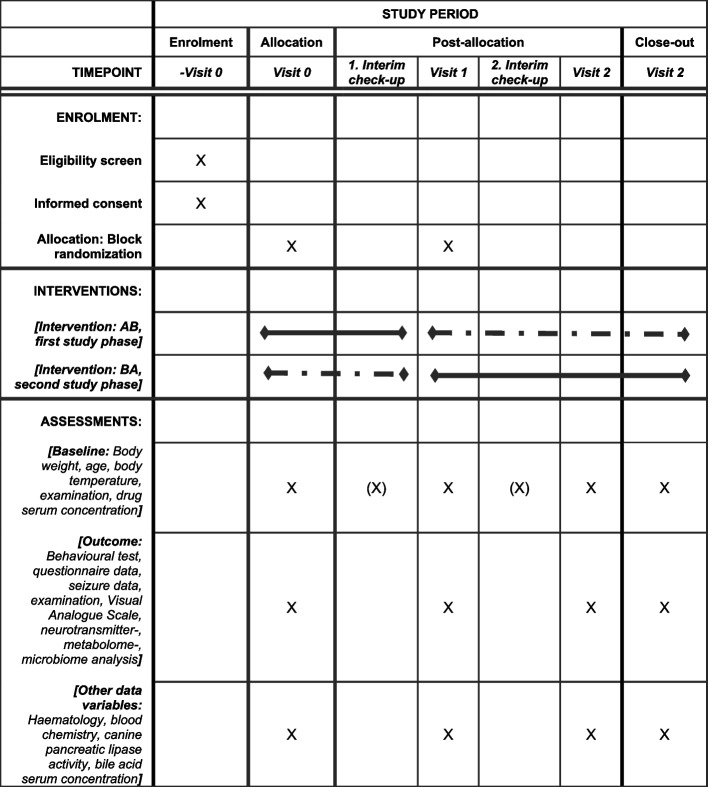
The SPIRIT (Standard Protocol Items: Recommendations for Interventional Trials) flow diagram. A schedule of enrolment, interventions, and assessments of the trial protocol

**Fig. 3:**
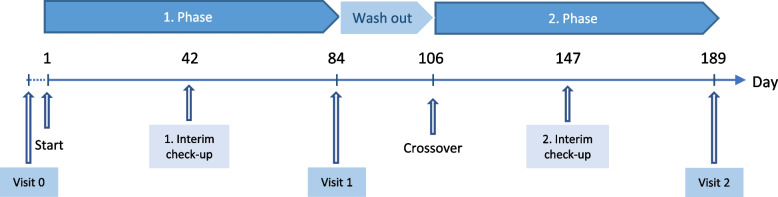
Study design of a six-month prospective, randomised, double-blinded, placebo-controlled, crossover, dietary trial. The aim of the current study is to investigate the influence of *Bifidobacterium longum* on the behaviour profile and secondarily on seizure semiology of 34 dogs. In the first phase each dog receives the allocated supplement (probiotic vs. placebo) alongside its normal diet for 3 months. The second phase starts after a wash out period of 3 weeks, followed by administering the other supplement (crossover) for another 3 months. Halfway through each study phase interim check-ups are conducted

Eligible dogs (*n* = 34) that match the selection criteria will be block randomised and assigned to their initial group, either the intervention group or the placebo group (Fig. 2). Due to the double-blinded study design, neither the owner nor the investigators know which group the dogs will be assigned to. In the first phase of the study, each dog will receive the allocated supplement alongside its normal diet for a period of 3 months (day 84 ± 2). In the second phase of the study, each participant will be moved (crossover) to the respective treatment group. A wash out period of 3 weeks (+ 21 days) is planned to reduce any possible carry-over effect. This period will not be included in the statistical analysis. The second phase of the study will last 3 months (day 84 ± 2). Throughout the whole study, each participant will be examined twice. At the beginning of the trial (Visit 0 = day 0) and after each supplementing period, all participants will undergo an on-site visit at the clinic (Visit 1 = day 84 ± 2, Visit 2 = day 189 ± 2). Halfway through each study phase, owners will be contacted by phone (1. Interim check-up = day 42 ± 2, 2. Interim check-up = day 147 ± 2). The interim check-ups are used to ensure compliance and to discuss any difficulties (Fig. 3). During enrolment and the entire study period, participating dogs will be medically attended by a neurologist of the TiHo. Clinical condition, blood parameters and long-term ASD serum concentration of participating dogs will be monitored at each on-site visit to ensure the highest standard of care. Owners and external veterinarians can contact the investigator directly via email at any time or the TiHo via phone, which will be forwarded to the investigator.

The owners of the participants are asked to keep the regular diet and long-term ASD treatment of the dog stable for the 6-month study period. Voluntary withdrawal from the study participation is possible at any time point. In case of severe worsening of clinical signs, the ASD serum concentration will be assessed and ASD dosage will be adjusted when required. In this case and if adjustment is not an option and a new long-term add-on ASD is mandatory, the trial participation will be terminated. A rescue therapy plan to treat acute cluster seizures or status epilepticus, will be given to the owners at the beginning of the trial. Rescue therapy with diazepam, midazolam, levetiracetam- or rivotril-pulse therapy administered by the owners is possible during the study. In the case of acute uncontrollable seizures, despite rescue therapy application, the emergency service at the TiHo is always available. In those extreme cases of epilepsy worsening, the seizures will be managed for the short term and it will be discussed with the owner if the trial can be continued. Human endpoints of the trial are firstly a severe worsening of clinical signs, considering the individual epilepsy course of each dog. The owner can decide at any given state to remove the dog from the study. Secondly, developing epilepsy-independent conditions requiring specific pharmacological or surgical interventions will lead to exclusion from the study. Thirdly, diet-associated gastrointestinal clinical signs not remitting after 6 days of treatment will end the participation When the dogs have received antibiotics, a regeneration period of 3 weeks is scheduled for the intestinal microbiome prior to the first visit. In case of administering another probiotic, there is a pre-wash out period of 3 weeks planned before the start of the first intervention period. Adverse events and additional medication during study participation must be carefully noted in a standardised diary by the owner (Additional file 3). The last criterion resulting in study exclusion is a lack of owner compliance with the experimental conditions.

The presented study protocol follows the Harmonised Animal Research Reporting Principles (HARRP), the Standard Protocol Items: Recommendations for Interventional Trials (SPIRIT) guidelines and the “Animal Research: Reporting of In Vivo Experiments” (ARRIVE) guidelines, where applicable (Additional files 4 and 5) [65–67].

### Baseline diet and probiotic supplement

The previous baseline diet of the dogs should be continued for the study period. The administration of treats is permitted if the treats and the feeding frequency do not differ between the two phases. The exact diet plan of the dogs is evaluated via the online questionnaires at each on-site visit, regarding feeding frequency, feeding type (commercial, home-made, subtypes), components, treats and supplements (Additional file 1).

The respective investigated study supplement is administered as one capsule daily for adult dogs in addition to their regular diet. Each capsule compromises a highly palatable powder which can be added to the baseline diet or in concentrated form to the dog’s bowl. The supplement of the intervention group contains *Bifidobacterium longum* and palatability enhancer, and the supplement of the placebo group contains solely palatability enhancer. At Visit 1 and Visit 2, the respective original package is collected and the number of remaining capsules inside are counted to monitor adherence to the study protocol.

### Study procedures

The owners will be contacted at specified time points during the ongoing study, with a tolerance of ±2 days:

- **Visit 0** (fist on-site visit): day 0 = before the beginning of the first study phase
  1. **Interim check-up** (fist telephone call): day 42 ± 2 = halfway through the first study phase
- **Visit 1** (second on-site visit): day 84 ± 2 = before the wash out period and the beginning of the second study phase
  2. **Interim check-up** (second telephone call): day 147 ± 2 = halfway through the second study phase
- **Visit 2** (third on-site visit): day 189 ± 2 = completion of the study

At each on-site visit, epilepsy progression and medical condition of the patient are assessed, additionally, further data are collected:

- Clinical and neurological examination
- Body weight recording
- Standardised seizure diary evaluation (seizure frequency, severity, subtype) [only at Visit 1 + Visit 2] (Additional file 3)
- Visual Analogue Scale assessment (ataxia, sedation, sleep, overall QoL)
- Blood analysis (haematology, blood chemistry, dynamic bile acid test [only at Visit 0], serum levels of the ASDs, serotonin serum level)
- Collection of fresh urine and faecal samples (neurotransmitter and microbiome analysis) [only at Visit 1 + Visit 2]
- Behavioural tests (exploratory behaviour, stranger-directed behaviour, fear and anxiety, noise sensitivity)
- Heart rate and heart rate variability recording during the behavioural tests
- Salivary cortisol determination before and after the behavioural tests

After each on-site visit, a standardised online questionnaire will be sent to the owner who retrospectively evaluates either the previous 6 months before study enrolment [Visit 0] or the previous 3 months of the respective study phase [Visit 1, Visit 2] (Additional file 1). The first questionnaire is part of the two-step recruitment process and will be completed by the owner prior to the first on-site visit [Visit 0] to ensure the study eligibility of the patient. The second and third questionnaires will be filled in following the regular study control appointments [Visit 1, Visit 2]. The dog owners gain access to the questionnaires via an online link sent by e-mail. To ensure the comparability of the collected data, the structure of the three questionnaires is similar, and they evaluate the following aspects: general information, seizure semiology, behavioural profile and QoL.

Concomitant treatments and additional medication (such as vaccines, endo- and ectoparasite prophylaxis) are documented during each phone call and appointment. The investigator records indications for treatment, product name, start and end date, dosage, application route and frequency. In exceptional cases, a dose adjustment of the currently applied ASDs or the administering an unavoidable treatment with antibiotics is tolerated to ensure animal welfare. These interventions result in a pausing of study participation, a prolongation of the corresponding study phase and will be considered in the statistical analysis. The beginning of a new long-term add-on ASD will result in study exclusion. Furthermore, an adverse event form is archived, when abnormal clinical signs occur during study participation, regardless of whether they can be linked to the dietary supplement or not. The adverse event form records duration in days, severity (mild, moderate, severe), detailed description, diagnosis, treatment requirement, causality and the first observation of the clinical signs. The occurrence of seizures is not classified as an adverse event and will be documented in the standardised seizure diary (Additional file 3).

### Specification of the study variables

#### Clinical and neurological examination, blood analysis

Comprehensive data on the patient’s health condition will be collected at every on-site visit. First of all, body weight, age and body temperature of each dog are acquired, followed by general clinical examination and neurological examination. After blood collection by venepuncture, the following parameters will be determined using laboratory diagnostics: haematology, blood chemistry, dynamic bile acid test (pre- [Visit 0, Visit 1, Visit 2], post-prandial [Visit 0]), serum levels of the ASDs (phenobarbital and potassium bromide) and serotonin serum level.

#### Behavioural comorbidities and quality of life

1. Questionnaires:

To evaluate the primary outcome of the study, changes in the behavioural profile of the dogs, a standardised online questionnaire will be sent after each visit and completed by the owner (Additional file 1). The questionnaire design is based on previously validated questionnaires:

- Attention Deficit Hyperactivity Disorder Rating Scale (ADHD RS) [55]: Four items of the ADHD RS are included in the questionnaire. These items rated on a four-point scale [1–4] evaluate attention deficit of the participants. A previous study has shown significantly higher scores in dogs suffering from benign juvenile epilepsy [56].
- Dog Personality Questionnaire (DPQ) [57]: To measure excitability and impulsivity of the dogs, five items of the DPQ are utilised. The rating scale range from 1 to 5. Jokinen, Tiira [56] found significantly higher scores in dogs suffering from benign juvenile epilepsy for the selected items, too.
- Canine Cognitive Dysfunction Rating Scale (CCDR) [58]: The CCDR determines cognitive changes and their progression. It comprises 13 questions on a rating scale from 1 to 5 and considers dog’s QoL and dog-owner relationship (focus on orientation problems, memory difficulties, apathy, olfactory impairment and locomotion). Scores with a threshold of > 50 indicate cognitive impairment and canine cognitive dysfunction.
- Canine Behavioural Assessment & Research Questionnaire (C-BARQ) [59]: In the C-BARQ, behaviour and temperament traits in dogs are analysed. It is used to investigate clinical effects of various treatments for behavioural problems. Owners rate in 68 statements, subdivided into 11 groups, on a scale of 0–4 how often the behaviour is shown (stranger-directed aggression, owner-directed aggression, dog-directed fear or aggression, stranger-directed fear, non-social fear, separation-related behaviour, pain sensitivity, attachment or attention-seeking behaviour, trainability, chasing, excitability).
- Evaluation of Quality of Life in Dogs with IE (EpiQoL) [68]: The EpiQoL examines physical, social and neurobehavioural aspects of dogs suffering from IE and their owners’ QoL. In this study, five groups of the original questionnaire will be included (“Adverse effects of antiepileptic drug (AED)”, “Restrictions on the carer’s life”, “Frustration over caring for a dog with IE”, “Owner distaste of AED adverse effects”, “Carer anxiety around the seizure event”). Statements are assessed using interval scoring on a scale from 1 to 5 and QoL improvement associated with therapeutic intervention can be determined (rating scale is modified from 0 to 5 for the adverse effects of AED group).

(2) Behavioural test:

To complete the behavioural profile analysis of the participants an additional behavioural test will be conducted at each of the on-site visits at the Small Animal Clinic of the University of Veterinary Medicine Hannover, Germany. The three-part behavioural test simulates day-to-day situations for dogs and is based on the modified separation and greeting test of Konok et al. [69], the open field test of Gruen et al. [70] and a modified version of Ainsworth’s strange situation test (ASST) from Pirrone et al. [71] and Palestrini et al. [72]. The standardised trial takes place in a video-monitored test room and is performed in the presence of the owner to rule out bias due to separation anxiety of the dog (exception: separation phase). The behavioural reactions recorded in the video clips will be analysed retrospectively, determining the activity, vocalisation, body posture and body language of the participants (distance covered in metres; frequency and intensity of behaviour (Likert-scale grading 1–10); occurrence of behaviour in percentage of entire test-time).

- Open field test (new environment): In the first part, exploratory behaviour in an unfamiliar room is assessed.
- Separation test and Stranger-directed-fear test: following a habituation phase, the interaction with a stranger is evaluated. In the separation phase, the dog is left alone in the room to record possible separation anxiety. In the final greeting phase, the owner enters the room again and greets his dog intensively.
- Open field test (noise): In the final part of the test, an audio recording of a thunderstorm is played (mean sound level 88 dB) to investigate noise-related anxiety or fear.

The measurement of supplementary physiological parameters complements the behavioural profile analysis to evaluate the stress response of the dogs during the behavioural test. For this purpose, salivary cortisol levels are determined at each visit before and after the behavioural test [73]. Saliva samples of the participants are obtained using Cortisol-Salivettes® (Sarstedt AG & Co. KG, Nümbrecht, Germany). Quantification of salivary cortisol concentration will be conducted utilising a competitive immunoassay: “Expanded Range High Sensitivity Salivary Cortisol Enzyme Immunoassay Kit” (“Salimetrics”/Biozol GmbH, Eching, Germany).

During the entire behavioural test, heart rate and heart rate variability of the participants will be measured via a Polar® H7 heart rate sensor (Polar Electro Oy, Kempele, Finland), validated for dogs in previous studies [74, 75]. Data are wirelessly transferred to the Elite HRV Tablet App (Elite HRV, Inc., Asheville, NC, USA) and assessed using the analysis software, Kubios HRV (Kubios Oy, Kuopio, Finland) [76]. Mean heart rate (increased by stress, excitement and activity) and heart rate variability (enables distinction between positive and negative stress) will be determined [72, 77, 78].

(3) Visual Analogue Scale (VAS):

In the presence of the investigator, the owner will subjectively assess four aspects impacting their dog’s QoL using VAS. Each aspect line is 0–100 mm long and the intersecting line set by the owner will be evaluated in percentage representing the restriction severity. The following aspects will be taken into consideration: ataxia (normal to animal cannot walk), sedation (normal to animal only sleeps), sleep (normal to severe trouble falling asleep/restless sleep/often awaken during sleep) and overall QoL (normal to euthanasia is requested).

#### Seizure semiology

For the entire trial period, specific seizure data of the participants (inclusive seizure triggers) will be documented by the owners in a standardised seizure diary (Additional file 3). The short-term efficacy of the intervention will be evaluated over the 3-month study phase. Only generalised seizures will be considered for efficacy assessment in the statistical analysis. The secondary outcome of this study is the treatment success of the probiotic intervention on seizure frequency. Treatment success in canine epilepsy is defined by the IVETF as being seizure free for a period lasting three times longer than the pretreatment interictal interval and at least 3 months, this being the primary therapy goal [79]. If seizures continue to occur, partial therapeutic success is the secondary goal, defined as prevention of cluster seizures or status epilepticus, relevant reduction in seizure frequency in consideration of the pretreatment seizure frequency and reduction in seizure severity [79]. Treatment success in this study is defined as as participants being seizure free throughout the respective study phase. Partial therapeutic success will be evaluated in dogs participating in the study for at least 3 months and receiving a consistent long-term ASD treatment for this phase. Participants will be categorised as “seizure free” with having no seizures throughout the respective study phase and as “responder” with a seizure frequency reduction of at least of 50% between both study phases.

Furthermore, the seizure diary is used to determine the seizure type, according to the definition of the IVETF consensus report [80] (Additional file 3). Generalised epileptic seizures affect both sides of the body because both cerebral hemispheres are involved [80]. Usually, the motor system is affected, with a combination of vegetative symptoms and always a loss of consciousness [80]. In contrast, focal seizures are limited to one cerebral hemisphere [80]. Cluster seizures are defined as more than one epileptic seizure within a 24-hour period and the regaining of consciousness between seizures [80]. A status epilepticus is a continuous epileptic seizure lasting longer than 5 minutes, or two or more epileptic seizures without regaining consciousness between seizures (for generalised convulsive seizures) [80].

#### Metabolome, microbiome and neurotransmitter evaluation

At the second and third on-site visit (Visit 1, Visit 2), fresh faecal and urine samples of the dogs collected by the owners will be required. These samples, including the blood samples will be stored for further microbiome, metabolome and neurotransmitter profile analysis.

The microbiome of the participants will be characterised and changes caused by probiotic supplementation will be evaluated at the Texas A&M College of Veterinary Medicine & Biomedical Sciences, TX, USA. Microbial deoxyribonucleic acid (DNA) is extracted from the faecal samples and analysed by quantitative polymerase chain reaction (qPCR) assay [81]. The assessment of the microbiome changes is performed via the qPCR-based dysbiosis index (DI) developed by AIShawaqfeh et al. 2017 [82]. The analysis focuses on eight bacterial groups, which are pathologically altered in diseased dogs (chronic inflammatory enteropathy) compared to healthy animals. *Faecalibacterium, Turicibacter, Escherichia coli, Streptococcus, Blautia, Fusobacterium, Clostridium hiranonis* and total bacteria could be identified as biomarkers for dysbiosis. The DI summarises the findings in one number and allows a reliable estimate of the presence of physiological microbial flora in the faeces (negative DI) or gives an indication of a dysbiosis (positive DI).

Blood samples are used for metabolome profiling and assessment of the probiotic influence on systemic metabolism of the participants. Analysis of 44 serum metabolites (creatinine, albumin, glycolysis-related metabolites, triglycerides, lipoprotein profiling, fatty acids, cholesterol and glycoprotein acetyls (systemic inflammation marker)), which serve as canine biomarkers, are determined by quantitative nuclear magnetic resonance (NMR) spectroscopy at PetMETA (PetBIOMICS Oy, Helsinki, Finland) [83]. Deviations in some metabolites (glutamine, γ-glutamyl glutamine, lipid and tryptophan metabolites) are associated with behavioural changes (anxiety related disorders/ attention-deficit/hyperactivity disorder [ADHD]) [84, 85]. Further metabolites (3-hydroxybutyrate, hexuronic acid, ribose, gluconic acid lactone) are associated with dysbiosis [86].

Quantification of urinary neurotransmitter levels (serotonin, histamine, glycine, phenylethylamine, dopamine, epinephrine, norepinephrine, glutamate, GABA) will be conducted utilising high-performance liquid chromatography triple-quadrupole mass spectrometry/mass spectrometry technology (Doctor’s Data, St. Charles, IL, USA). Recent studies have shown altered urinary neurotransmitter patterns of dogs suffering from IE and associations with the treatment response of drug-resistant dogs [87] (unpublished data).

### Data analysis

The statistical software Prism® (GraphPad Software, Inc., San Diego, CA, USA) and SAS® (SAS Institute, Inc., Cary, NC, USA) are used for data analysis and graphic presentation; afterwards, to data acquisition is completed. The significance p-level for the results will be set at < 0.05.

To assess the efficacy of the probiotic in comparison to the placebo supplement, the data will be categorised into selected study variables and the following statistical analysis will be conducted, adhering to previous studies based on a similar study design (Additional file 6) [63, 64]. For each group (probiotic vs. placebo), individual parameters will be registered and represented graphically. Descriptive statistics will be performed for continuous variables of each group. The mean and standard error will be calculated for each group if data are normally distributed, which will then be evaluated via histograms and using the Kolmogorov-Smirnov-test. Discrete or categorical values will be presented in tabular form and a frequency table will be created for each group. The results will be graphically displayed in bar or pie charts for each group.

The seizure frequency will be assessed by counting the number of seizures per month. The seizure severity will be evaluated using the McNemar test to compare the occurrence of cluster seizures between study periods. The match-paired Student’s t-test for parametric data and the Wilcoxon matched-pairs signed rank test for non-parametric data will be used to compare the dietary supplement groups. Pearson’s correlation coefficient analysis for parametric and Spearman’s test for non-parametric data will be applied to determine the relationship between two variables (e.g. seizure frequency and age). For individual data sets, multiple comparison correction will be considered if necessary. The data analysis will be a combination of univariate and multivariate analyses. A mixed effect model will potentially be applied and statistical analysis adjusted when required.

## Discussion

The collected data of the planned crossover study will provide valuable results on whether supplementation with the probiotic *Bifidobacterium longum* reduces behavioural comorbidities and improves the seizure semiology due to the partially overlapping pathomechanisms in drug-resistant dogs suffering from IE. If the clinical and statistical results indicate a positive effect, this innovative treatment can become a component of a multimodal epilepsy therapy, enhancing drug-sensitivity, improving comorbidities and overall QoL in dogs with IE.

This study is designed as a 6-month prospective, block randomised, double-blinded, placebo-controlled, crossover, dietary trial. It will be conducted in consideration of modern veterinary and modified human high-quality standards for epilepsy medication trials [88–91]. Therefore, common biasing effects in epilepsy trials, like the regression-to-the mean effect, the placebo effect and the observer effect, should be limited to a possible minimum [92–94]. This will ensure a high validity and accuracy of the acquired results, enable a representative nature of the efficacy of *Bifidobacterium longum* as add-on supplement for dogs suffering from IE and associated comorbidities.

The regression to the mean (RTM) effect is a group phenomenon in clinical studies, appearing when a variable of a subgroup is extreme at its first measurement and closer to the mean of the overall population at its second one or vice versa [93, 95]. The participants in this intervention trial represent an extreme group of the IE population, with dogs suffering from a severe phenotype of IE (high seizure frequency, drug-resistant to at least one ASD) and additional behavioural abnormalities. IE is a heterogeneous and unpredictable condition in humans and dogs [89, 96]. Exacerbation, as well as spontaneous remission of the disease may occur over the 6-month study period by chance, regardless of treatment efficacy [89, 96]. Additionally, the urge to participate for an owner in an epilepsy trial is higher when the course of the disease is particularly bad. Due to this factor and the undulatory progression of IE, the RTM during participation is possible, especially in patients, which barely met the required seizure frequency per month [89]. By certain adjustments in study design the RTM effect can be diminished [95]. One of those is assessing multiple baseline measurements to identify eligible trial participants [95]. Thereby the real mean and intra-individual variability of potential candidates is appraised [95]. Including patients in the study with lower value variability lowers the RTM effect [95]. Therefore, a 6-month retrospective seizure diary will be reviewed to evaluate suitability of long-term seizure frequency and individual course of disease before enrolment in the current study. Another method to address the RTM effect is the random allocation of participants to comparison groups [95]. Including a placebo group, identically influenced by the RTM effect, enables comparison of the mean change between both groups (intervention vs. placebo) and an assessment of treatment efficacy [95]. This method is taken into account as well, by designing a block randomised placebo-controlled crossover trial for the current study.

Historically, the placebo effect is present in every field of human medicine and is especially pronounced in psychiatric disorders like depression and anxiety [97, 98]. Kirsch et al. estimated that a significant proportion of treatment success in human depression can be attributed to the placebo effect [99]. The mechanism of the phenomenon is currently unknown, but it is assumed that positive expectations of the participants have an influence on neurobiology, resulting in modified treatment efficacy [100]. In veterinary medicine, the placebo effect is recognised as well, but a limited number of studies evaluating the subject exist [101]. The available literature describes caregiver placebo effects as a response to different treatment approaches in orthopaedic diseases of small animals [102–104]. In canine epilepsy trials, the placebo effect occurred in 29% of the dogs, showing a reduction in seizure frequency after administering a placebo, which is similar to the effect seen in an uncontrolled trial in drug-resistant canine patients [92]. To preclude a biasing impact on the important outcome measures, seizure frequency and behaviour (e.g. anxiety), this study is designed as a placebo-controlled trial. In psychiatric disorders of humans, the placebo effect arises earlier than the actual intervention effect, tends to interrupt more quickly and is hard to maintain over a longer period of time [105]. In clinical epilepsy trials of human medicine, it occurred more frequently with a recent onset of the disease and was less common in severe epilepsy phenotypes of drug-resistant patients [106, 107]. Consequently, the inclusion criteria will minimise the placebo effect in addition to the placebo-controlled study design and the long study period will enable a distinction of the intervention efficacy.

The Hawthorne effect arises when individuals behave differently with the awareness of being observed during study participation [108]. Due to a poor owner compliance rate of 56% in canine epilepsy, the impact of the Hawthorne effect might be significant [109]. Over the course of the study, the effect could have a primary impact on the routine ASD administration by the owner and subsequently an impact on seizure semiology and the behaviour of the dogs, misleading to an incorrect assessment of the intervention efficacy. To exclude the Hawthorne effect, a previous period of 6 months of the seizure semiology will be retrospectively analysed. Moreover, at the first on-site visit (Visit 0), data acquisition will be conducted before the start of the first observed intervention phase.

In a placebo-controlled crossover study, each participant is assigned to both groups (intervention- and placebo group) and crosses over to the other group after an intervening wash out period [94]. Thereby, each dog receives the probiotic intervention and the patient’s individual course of epilepsy can be taken into account for evaluating treatment efficacy [96]. The participants serve as their own controls, which reduces data variance and enables a smaller study population [110].

A wash out period of 3 weeks will be considered as sufficient to eliminated potential probiotic remains and resulting effects. Permanent changes in the microbiome of small animals and humans caused by probiotic administration are proven to be unlikely [111–114]. In dogs and cats, the colonisation of the gut by given probiotics is typically temporary [115]. The effect of the probiotic is mediated by the production of beneficial metabolites during host passage [115]. Two canine studies demonstrated no significant microbiome changes after the cessation of probiotic supplementation in evaluated faecal sample [111, 112]. In those studies, follow-up faecal samples were analysed either 3 weeks or 6 weeks after former terminated administering of probiotics, also including a certain *Bifidobacterium longum* strain (NCIMB 30179) [111, 112]. Furthermore, the applied three-week wash out period in the current study is in accordance with a previous crossover trial, which investigated the similar strain of *Bifidobacterium longum* in dogs (BL999) [47]. Identical to the results of the canine studies, the majority of human studies also showed limited lasting microbiome changes after discontinuing probiotic intake. The supplemented bacteria strains, also including bifidobacteria, were generally detectable for less than 2 weeks [113, 114, 116–119]. However, in some individuals a more permanent colonisation of the gut occurred [117, 120]. To author’s knowledge such individual differences were not detected in dogs yet. Provided evidence demonstrated that a carry-over effect of the probiotic beyond the wash out is unlikely. Though, by comparing data of dogs receiving the probiotic intervention in the first study phase with those receiving it in the second study phase, a potential biasing effect of the crossover design on the gained results may be revealed.

Blinding is a method to ensure the validity of study results and avoid subjective bias during data evaluation [94]. This trial is designed as a double-blinded study; neither the owner nor the investigator knows the first and second assigned group of the dogs, to avert prejudiced treatment expectations on both sides [94].

Randomised controlled studies are commonly used in clinical trials in human and veterinary medicine and provide the most credible outcome among all research types [94]. Participants are allocated to their treatment group by chance at the beginning of the study to minimise selection bias [94]. This trial uses a randomised block design and each dog is assigned to their respective rotation block at the timepoint of study enrolment (1st intervention group, 2nd placebo group = 1st block; or vice versa = 2nd block). Block randomisation ensures an equal number of individuals are assigned to each rotation block and increases the power of the study results, especially in a small sample size [121, 122]. The randomisation process is conducted via “random.org” (Randomness and Integrity Services Ltd., Dublin, Ireland) by an uninvolved, unblinded scientist, and block assignment is revealed at the end of the data acquisition.

Planning a clinical trial, the dropout rate must be taken into consideration. Dropout is commonly used as an outcome measure, reflecting drug tolerability, adverse effects and a lack of compliance [123]. Clinical trials investigating treatment of epilepsy often have a higher dropout rate due to their study design, e.g. long follow-up periods, placebo control groups and fixed dosing [123, 124]. Previous dietary intervention epilepsy trials with a similar study design based on a study population of 21 dogs provided significant results for an antiepileptic treatment effect and an influence on the behavioural profile [50, 63]. A previous study with a *Bifidobacterium longum* probiotic has shown positive effects in 24 dogs without epilepsy [47]. Regarding former studies of LAW et al. [63] and BERK et al. [64], we consider an approximate dropout rate of 30%.

Despite consideration of modern human and veterinary guidelines for designing epilepsy trials, not all sources of variability can be eliminated in clinical studies and a few limitations remain.

Following the Consolidated Standards of Reporting Trials (CONSORT) statement, we are aiming to provide a per-protocol (PP) analysis and an intention to treat analysis (ITTA) [125]. In the PP analysis, all outcomes of participants are assessed that complied with the study protocol as planned and received the intervention through the complete scheduled study period [126]. Unavoidably, the PP analysis creates a subgroup, and the comparability of the enrolled patients, as well as the effect of the randomisation may be lost [126]. Therefore, an ITTA should also be conducted in prospective randomised trials [127, 128]. For an ITTA, all randomised participants are evaluated in statistical analysis according to their assigned treatment group to prevent a positive bias, which would result in the exclusion of non-responding patients [128]. In a previous study based on a similar trial design, patients mainly dropped out during the first study period [63]. According to the design, no outcome measures could be acquired for these participants and were not available for subsequent ITTA. In the case of withdrawing from this study during the first study period, the owners will be given the option to switch the patient to the second study period to be able to perform an ITTA. Additionally, a final appointment will be made for any dropout. These records will reflect daily clinical practice and guarantee a high study power [126]. In every case, the detailed reason for dropping out will be documented, especially if a final appointment does not take place. The statistical imputation method for outcome measures will not be used, because the missing outcome is not predictable. An assumption of the missing values will bias the results in one way or another.

The secondary outcome of the study (seizure frequency) and seizure type of the dogs will be evaluated, based on owner reports, documented in seizure diaries. This could be a possible limitation of the trial. The compliance for daily seizure records in the diary is essential. Unfortunately, seizure diaries are often retrospectively completed prior to a study visit and not in real time, resulting in an inaccurate recollection of the seizure frequency and type [89]. Furthermore, owners cannot always distinguish the seizure types of their dogs correctly and the perception of focal seizures is lower than that of generalised seizures [129]. Consequently, only generalised seizures will be considered for efficacy assessment in this study. In a human epilepsy study, exclusive consideration of generalised seizures caused lower placebo response rates [130]. To improve seizure records in future epilepsy trials in human and veterinary medicine, technical devices, which detect and log seizures are deemed as a valuable option [89, 131]. Currently, the overall sensitivity of the devices for detecting of generalised seizures in dogs is low; therefore, seizure diaries will still be used in this study [132].

One disadvantage of the crossover design may inevitably influence the results of the study; depending on owners’ perception of the treatment efficacy of the first study phase, expectations for the second study phase may evoke in a particular direction [110]. Nevertheless, the crossover design has been chosen for this study because the positive effects predominate.

Although all dogs must meet the same study inclusion criteria, a heterogeneous group will be enrolled, with differences in their baseline diet and their ASD treatment. On the one hand, this heterogeneity could have an unpredictable influence on pharmacokinetic and pharmacodynamic interactions and subsequently on intervention efficacy, as is the case in all add-on epilepsy trials [89]. On the other hand, a heterogeneous group will limit the artificiality of the selected subgroup, creating a representative outcome for the general patient population and clinical usage [90]. This may not only indicate efficacy but also effectiveness of *Bifidobacterium longum* as add-on supplement for dogs suffering from IE and its comorbidities [90].

## Supplementary Information

**Additional file 1.** Standardised online questionnaire. The questionnaire is evaluating the following aspects: general information (signalment, diet, training, diagnostics and treatment of epilepsy), seizure semiology, behavioural profile and quality of life. The questionnaire is based on previously validated owner-completed behavioural questionnaires: Attention Deficit Hyperactivity Disorder Rating Scale (ADHD RS) [55], Dog Personality Questionnaire (DPQ) [57], Canine Cognitive Dysfunction Rating Scale (CCDR) [58], Canine Behavioural Assessment & Research Questionnaire (C-BARQ) [59], Evaluation of Quality of Life in Dogs with IE (EpiQoL) [68]. Gained data is used to assess the initial study eligibility, to acquire retrospective data of the baseline period prior study entry and prospective data of the intervention and placebo period during trial participation.

**Additional file 2.** Owner information sheet. Providing detailed information about the aim and procedure of the intervention study.

**Additional file 3.** Standardised seizure diary. Owner recordings of seizure frequency, severity, subtype, seizure triggers, antiseizure drug administration, probiotic administration, rescue therapy, adverse events and additional medication.

**Additional file 4.** The SPIRIT (Standard Protocol Items: Recommendations for Interventional Trials) item checklist.

**Additional file 5.** The Animal Research: Reporting of In Vivo Experiments” (ARRIVE) guidelines 2.0: author checklist.

**Additional file 6: Table 1.** Study variables: at each on-site visit, data of primary outcome (behaviour), secondary outcome (seizure semiology) as well as further data of the participants are collected. The table provides a detailed overview of categorising the collected data into preselected study variables and premeditated statistical analysis to evaluate the efficacy of the probiotic (*Bifidobacterium longum*) in comparison to the placebo supplement. In addition, comparison groups are created of the study phase (phase 1 vs. phase 2), study supplement (probiotic vs. placebo), responder rate (seizure frequency reduction of at least 50%), seizure semiology (seizure type: occurrence of generalised and focal seizures; history/occurrence of cluster seizures, history/occurrence of status epilepticus) and other relevant factors of the questionnaire and behavioural test.

## Data Availability

The collected data during this study will be compiled, stored and used by the Department of Small Animal Medicine and Surgery, University of Veterinary Medicine Hannover for research, lecture and publication purposes. All personal information will be treated securely, confidentially and will be anonymised beforehand, following the European General Data Protection Regulation 2016/679. Datasets are not applicable to this article, because it describes only the study design. The datasets and data analysis will be published separately.

## Abbreviations

QoL: Quality of life
GABA: γ-aminobutyric acid
CNS: Central nervous system
SCFAs: Short-chain fatty acids
ASD/s: Antiseizure drug/s
FMT: Faecal microbiota transplantation
MCT: Medium chain triglyceride
IE: Idiopathic epilepsy
TiHo: Small Animal Clinic of the University of Veterinary Medicine Hannover, Germany
IVETF: International veterinary epilepsy task force
MRI: Magnetic resonance imaging
HARRP: Harmonised animal research reporting principles
SPIRIT: Standard Protocol Items: “Recommendations for Interventional Trials
ARRIVE: Animal Research: Reporting of In Vivo Experiment”
ADHD RS: Attention-deficit/hyperactivity disorder rating scale
DPQ: Dog Personality Questionnaire
CCDR: Canine cognitive dysfunction rating scale
C-BARQ: Canine behavioural assessment & research questionnaire
EpiQoL: Evaluation of quality of life in dogs with idiopathic epilepsy
AED: Antiepileptic drug
ASST: Ainsworth’s strange situation test
VAS: Visual analogue scale
DNA: Deoxyribonucleic acid
qPCR: Quantitative polymerase chain reaction
DI: Dysbiosis index
NMR: Nuclear magnetic resonance
ADHD: Attention-deficit/hyperactivity disorder
RTM: Regression to mean
CONSORT: Consolidated Standards of Reporting Trials
PP: Per-protocol
ITTA: Intention to treat analysis
