# Supplementary material for: A six-month prospective, randomised, double-blinded, placebo-controlled, crossover, dietary trial design to investigate the potential of psychobiotics on seizure semiology and comorbidities in canine epilepsy: study protocol
Source: BMC Vet Res. 2023 Mar 3;19:57. doi: 10.1186/s12917-023-03609-0 (PMC9983181; doi:10.1186/s12917-023-03609-0)
Supplement: Supplementary file 1 — Additional file 1. Standardised online questionnaire. The questionnaire is evaluating the following aspects: general information (signalment, diet, training, diagnostics and treatment of epilepsy), seizure semiology, behavioural profile and quality of life. The questionnaire is based on previously validated owner-completed behavioural questionnaires: Attention Deficit Hyperactivity Disorder Rating Scale (ADHD RS) [55], Dog Personality Questionnaire (DPQ) [57], Canine Cognitive Dysfunction Rating Scale (CCDR) [58], Canine Behavioural Assessment & Research Questionnaire (C-BARQ) [59], Evaluation of Quality of Life in Dogs with IE (EpiQoL) [68]. Gained data is used to assess the initial study eligibility, to acquire retrospective data of the baseline period prior study entry and prospective data of the intervention and placebo period during trial participation. [file 12917_2023_3609_MOESM1_ESM.pdf]

BL999-Studie: Umfrage zum Hundeverhalten und zur Lebensqualität (C1)

Sehr geehrte Damen und Herren,

vielen Dank, dass Sie sich für die Studienteilnahme entschieden haben.

Mein Name ist Teresa Schmidt und ich arbeite als Doktorandinnen an der Klinik für Kleintiere der Stiftung Tierärztliche Hochschule Hannover unter der Leitung von Professor Dr. Volk in der Neurologie.

In dieser Studie wird sich mit dem Verhalten von Hunden, sowie der Entwicklung neuer Therapiemöglichkeiten beschäftigen. Dazu benötige ich Ihre Unterstützung, denn durch den täglichen Umgang mit Ihrem Tier können Sie das Verhalten am besten beurteilen.

Mit Ihrer Hilfe können neue Erkenntnisse in der Kleintiermedizin gewonnen werden, die zur Verbesserung und Entwicklung von neuen Therapiemethoden und der Vorbeugung von neurologischen Erkrankungen des Hundes beitragen.

Ich freue mich sehr, dass Sie sich die Zeit nehmen, diese Umfrage zu beantworten. Der Zeitaufwand beträgt ca. 30 Minuten. Ihre Antworten werden automatisch über das Programm LimeSurvey ausgewertet und die Ergebnisse werden händisch anhand der ermittelten Scores kategorisiert.

Sollten Sie Fragen bezüglich des Inhalts haben, können Sie mich gerne per E-Mail kontaktieren.

Vielen Dank,

Teresa Schmidt

E-Mail: Teresa.Schmidt@tiho-hannover.de (mailto:Teresa.Schmidt@tiho-hannover.de)

Mit dem Öffnen der Umfrage willigen Sie in die Verarbeitung Ihrer Daten entsprechend den Datenschutzhinweisen ein.

In dieser Umfrage sind 59 Fragen enthalten.

Allgemeine Angaben

In der Umfrage wird aus Gründen der besseren Lesbarkeit von dem Maskulinum "der Hund" gesprochen. Weibliche Hunde werden dabei selbstverständlich und ausdrücklich mit gemeint.

Bitte geben Sie Ihre Teilnehmernummer an. *

❗ Bitte wählen Sie eine der folgenden Antworten:
Bitte wählen Sie nur eine der folgenden Antworten aus:

- ○ 1
- ○ 2
- ○ 3
- ○ 4
- ○ 5
- ○ 6
- ○ 7
- ○ 8
- ○ 9
- ○ 10
- ○ 11
- ○ 12
- ○ 13
- ○ 14
- ○ 15
- ○ 16
- ○ 17
- ○ 18
- ○ 19
- ○ 20
- ○ 21
- ○ 22
- ○ 23
- ○ 24
- ○ 25
- ○ 26
- ○ 27

- ◯ 28
- ◯ 29
- ◯ 30
- ◯ 31
- ◯ 32
- ◯ 33
- ◯ 34
- ◯ 35
- ◯ 36
- ◯ 37
- ◯ 38
- ◯ 39
- ◯ 40
- ◯ 41
- ◯ 42
- ◯ 43
- ◯ 44
- ◯ 45
- ◯ 46
- ◯ 47
- ◯ 48
- ◯ 49
- ◯ 50
- ◯ 51
- ◯ 52
- ◯ 53
- ◯ 54
- ◯ 55
- ◯ 56
- ◯ 57
- ◯ 58

- ◯ 59
- ◯ 60
- ◯ 61
- ◯ 62
- ◯ 63
- ◯ 64
- ◯ 65
- ◯ 66
- ◯ 67
- ◯ 68
- ◯ 69
- ◯ 70
- ◯ 71
- ◯ 72
- ◯ 73
- ◯ 74
- ◯ 75
- ◯ 76
- ◯ 77
- ◯ 78
- ◯ 79
- ◯ 80
- ◯ 81
- ◯ 82
- ◯ 83
- ◯ 84
- ◯ 85
- ◯ 86
- ◯ 87
- ◯ 88
- ◯ 89

- ◯ 90
- ◯ 91
- ◯ 92
- ◯ 93
- ◯ 94
- ◯ 95
- ◯ 96
- ◯ 97
- ◯ 98
- ◯ 99
- ◯ 100

Ihre Teilnehmernummer wurde Ihnen zu Beginn der Studie von den Studienleitern mitgeteilt.

Wie lautet der Name Ihres Hundes? *

Bitte geben Sie Ihre Antwort hier ein:

Bitte geben Sie das Alter Ihres Hundes an. *

❗ Bitte wählen Sie eine der folgenden Antworten:
Bitte wählen Sie nur eine der folgenden Antworten aus:

- ○ <1
- ○ 1
- ○ 2
- ○ 3
- ○ 4
- ○ 5
- ○ 6
- ○ 7
- ○ 8
- ○ 9
- ○ 10
- ○ 11
- ○ 12
- ○ 13
- ○ 14
- ○ 15
- ○ 16
- ○ 17
- ○ 18
- ○ >18

Die Angabe des Alters erfolgt in Jahren.

Welches Geschlecht hat Ihr Hund? *

Bitte wählen Sie eine der folgenden Antworten:
Bitte wählen Sie nur eine der folgenden Antworten aus:

- Männlich unkastriert
- Weiblich unkastriert
- Männlich kastriert
- Weiblich kastriert

Zu welcher Rasse gehört Ihr Hund? *

❗ Bitte wählen Sie eine der folgenden Antworten:
Bitte wählen Sie nur eine der folgenden Antworten aus:

- ◯ Australian Shepherd
- ◯ Beagle
- ◯ Belgischer Schäferhund
- ◯ Berner Sennenhund
- ◯ Bolonka Zwetna
- ◯ Border Collie
- ◯ Cane Corso
- ◯ Chihuahua
- ◯ Dackel
- ◯ Deutsche Dogge
- ◯ Deutscher Boxer
- ◯ Deutscher Schäferhund
- ◯ Deutscher Spitz
- ◯ Dobermann
- ◯ Englische Bulldogge
- ◯ Französische Bulldogge
- ◯ Golden Retriever
- ◯ Havaneser
- ◯ Jack Russell Terrier
- ◯ Labrador
- ◯ Magyar Vizsla
- ◯ Malteser
- ◯ Mischling
- ◯ Mops
- ◯ Old English Bulldog
- ◯ Pudel
- ◯ Rhodesian Ridgeback

◯ Rottweiler

◯ Shih Tzu

◯ Siberian Husky

◯ Yorkshire Terrier

◯ Sonstige Rassen

Sollte die Rasse Ihres Hundes nicht aufgeführt sein, wählen Sie bitte "Sonstige Rassen" aus.

Wenn sich Ihr Hund nicht eindeutig einer Rasse zuordnen lässt, wählen Sie bitte "Mischling" aus.

Wie schwer ist Ihr Hund? (in kg)

❗ In dieses Feld dürfen nur Zahlen eingegeben werden.
Bitte geben Sie Ihre Antwort hier ein:

kg

Beispiel: 5,3 kg

Angaben zur Fütterung

Wie oft füttern Sie Ihren Hund?

❗ Bitte wählen Sie eine der folgenden Antworten:
Bitte wählen Sie nur eine der folgenden Antworten aus:

◯ Einmal täglich

◯ Zweimal täglich

◯ Dreimal täglich

◯ Ad libitum (Futter steht durchgehend zur freien Verfügung bereit)

Was füttern Sie Ihrem Hund? *

❗ Bitte wählen Sie die zutreffenden Antworten aus:
Bitte wählen Sie alle zutreffenden Antworten aus:

- [ ] Nassfutter (Dose)
- [ ] Nassfutter (Beutel)
- [ ] Trockenfutter (Alleinfuttermittel)
- [ ] Trockenfutter (Mischung)
- [ ] Therapeutische / Verschriebene / Tierärztliche Diät
- [ ] Selbstgekochtes Futter
- [ ] Rohfutter (BARF)
- [ ] Vegetarisches Futter
- [ ] Belohnungen (Hundeleckerchen, z.B. Kekse, Denta-Sticks)
- [ ] Belohnungen (menschliche Nahrung, z.B. Speisereste)
- [ ] Sonstiges: ____________

Wenn Sie ein anderes Futtermittel anbieten, dann ergänzen Sie es bitte unter "Sonstiges".

Wenn Sie eines der folgenden Futtermittel anbieten, beschreiben Sie bitte genau, worum es sich handelt. *

Kommentieren wenn eine Antwort gewählt wird
Bitte wählen Sie die zutreffenden Punkte aus und schreiben Sie einen Kommentar dazu:

☐ Therapeutische / Verschriebene / Tierärztliche Diät

☐ Selbstgekochtes / Vegetarisches Futter

☐ Rohfutter

☐ Im Handel erhältliches Nass- oder Trockenfutter

Bei einem im Handel erhältlichen Futtermittel geben Sie bitte den Markennamen an.

Bzw. bei selbstgekochtem Futter: z.B. Hühnchen mit Reis

Wie häufig geben Sie Ihrem Hund Snacks oder Belohnungen?

Bitte wählen Sie eine der folgenden Antworten:
Bitte wählen Sie nur eine der folgenden Antworten aus:

◯ Täglich
◯ Mehrmals die Woche
◯ Mehrmals im Monat
◯ Nie

Welche Ergänzungsfuttermittel geben Sie Ihrem Hund und wie häufig verabreichen Sie diese?

Bitte wählen Sie die zutreffende Antwort für jeden Punkt aus:

	nie	weniger als einmal im Monat	monatlich	wöchentlich	täglich
**Vitamine**	○	○	○	○	○
**Glucosamine und/ oder Chondroitin**	○	○	○	○	○
**Fettsäuren oder Öle**	○	○	○	○	○
**Lebertran**	○	○	○	○	○
**Mittelkettige Fettsäuren**	○	○	○	○	○
**Epitaur ®**	○	○	○	○	○
**Mariendistel**	○	○	○	○	○
**Andere**	○	○	○	○	○

Andere bitte hier benennen:

Bitte geben Sie Ihre Antwort hier ein:

Angaben zum Training

Wurde mit Ihrem Hund eine Welpenschule/Welpengruppe besucht?

❗ Bitte wählen Sie eine der folgenden Antworten:
Bitte wählen Sie nur eine der folgenden Antworten aus:

- ◯ Ja
- ◯ Nein

Wie viele Stunden nahm Ihr Hund ca. an der Welpenschule/Welpengruppe teil?

Beantworten Sie diese Frage nur, wenn folgende Bedingungen erfüllt sind:
Antwort war 'Ja' bei Frage '13 [t1]' (Wurde mit Ihrem Hund eine Welpenschule/Welpengruppe besucht?)

❗ Bitte wählen Sie eine der folgenden Antworten:
Bitte wählen Sie nur eine der folgenden Antworten aus:

- ◯ 1-10 Stunden
- ◯ 11-20 Stunden
- ◯ über 20 Stunden

An welchem Training hat Ihr Hund teilgenommen? *

Bitte wählen Sie die zutreffende Antwort für jeden Punkt aus:

	Keine Teilnahme	Teilgenommen, aber aufgehört	Ja, immer noch aktiv
**Obedience**	◯	◯	◯
**Agility**	◯	◯	◯
**Schutzhund**	◯	◯	◯
**Jagen**	◯	◯	◯
**Training zuhause**	◯	◯	◯
**Sonstiges**	◯	◯	◯

Bitte kreuzen sie zutreffendes an!

Bitte tragen Sie hier das "sonstige" Training ein.

Beantworten Sie diese Frage nur, wenn folgende Bedingungen erfüllt sind:
Antwort war 'Teilgenommen, aber aufgehört' *oder* 'Ja, immer noch aktiv' bei Frage '15 [t2]' (An welchem Training hat Ihr Hund teilgenommen? (Sonstiges))

Bitte geben Sie Ihre Antwort hier ein:

Wurde mit Ihrem Hund im Bezug auf die oben genannten Trainingsarten eine Prüfung abgelegt? Wenn ja, welche?

Bitte geben Sie Ihre Antwort hier ein:

z.B. Begleithundeprüfung

Abfrage Epilepsie

Leidet Ihr Hund an epileptischen Anfällen? *

Bitte wählen Sie nur eine der folgenden Antworten aus:

- Ja
- Nein

Epileptische Anfälle sind definiert als zwei oder mehr Anfälle, die jeweils 24 Stunden auseinander liegen.

Weiterführende Fragen zur Epilepsie I/III

Mit welchem Alter trat der erste epileptische Anfall auf? *

Beantworten Sie diese Frage nur, wenn folgende Bedingungen erfüllt sind:
Antwort war 'Ja' bei Frage '18 [e1]' (Leidet Ihr Hund an epileptischen Anfällen?)

❗ Bitte wählen Sie eine der folgenden Antworten:
Bitte wählen Sie nur eine der folgenden Antworten aus:

- ○ <1
- ○ 1
- ○ 2
- ○ 3
- ○ 4
- ○ 5
- ○ 6
- ○ 7
- ○ 8
- ○ 9
- ○ 10
- ○ 11
- ○ 12
- ○ 13
- ○ 14
- ○ 15
- ○ 16
- ○ 17
- ○ 18
- ○ >18

Die Angabe des Alters erfolgt in Jahren.

Wie viele Anfälle hatte Ihr Hund schätzungsweise in den vergangenen 3 Monaten? *

Bitte geben Sie Ihre Antwort hier ein:

Welche der folgenden Untersuchungen wurden im Falle Ihres Hundes durchgeführt, um die Epilepsie zu diagnostizieren und welche Untersuchungsergebnisse wurden ermittelt? *

Bitte wählen Sie die zutreffende Antwort für jeden Punkt aus:

	normal	abnormal	nicht durchgeführt
**Blutuntersuchung (Blutbild, Blutchemie, Gallensäure usw.)**	○	○	○
**Harnuntersuchung**	○	○	○
**Magnetresonanztomographie (MRT) des Kopfes**	○	○	○
**Computertomographie (CT) des Kopfes**	○	○	○
**Entnahme und Untersuchung der Gehirn- und Rückenmarksflüssigkeit**	○	○	○

Bitte erläutern Sie die "abnormalen" Befunde im unten stehenden Kommentarfeld ausführlicher.

Sollten Untersuchungen durchgeführt worden sein, über deren Details Sie sich unsicher sind, geben Sie im Kommentarfeld bitte "unsicher" an.

Bitte geben Sie Ihre Antwort hier ein:

Steht Ihr Hund aktuell unter antiepileptischer Dauertherapie? *

❗ Bitte wählen Sie eine der folgenden Antworten:
Bitte wählen Sie nur eine der folgenden Antworten aus:

- ◯ Ja
- ◯ Nein

Erhält Ihr Hund aktuell eines oder mehrere der folgenden Medikamente?

Beantworten Sie diese Frage nur, wenn folgende Bedingungen erfüllt sind:
Antwort war 'Ja' bei Frage '23 [ep3]' (Steht Ihr Hund aktuell unter antiepileptischer Dauertherapie?)

❗ Bitte wählen Sie die zutreffenden Antworten aus:
Bitte wählen Sie alle zutreffenden Antworten aus:

- ☐ Imepitoin (Pexion)
- ☐ Phenobarbital (Epiphen, Phenoleptil, Luminal, Luminaletten)
- ☐ Kaliumbromid (Epilease, Libromide, Dibromide)
- ☐ Levetiracetam (Keppra)
- ☐ Zonisamid
- ☐ Gabapentin
- ☐ Diazepam
- ☐ Pregabalin
- ☐ Chlorazepat
- ☐ Felbamat
- ☐ Phenytoin
- ☐ Lamotrigin
- ☐ Tiagabin
- ☐ Vigabatrin
- ☐ Sonstige: ________

Wie ist die Dosierung der jeweiligen Medikamente?

- Häufigkeit der täglichen Gabe, Tablettenmenge und Dosierung

Beantworten Sie diese Frage nur, wenn folgende Bedingungen erfüllt sind:
Antwort war 'Ja' bei Frage '23 [ep3]' (Steht Ihr Hund aktuell unter antiepileptischer Dauertherapie?)

Bitte geben Sie Ihre Antwort hier ein:

z.B.: 2 x tgl. 1 Tablette Levetiracetam 250 mg

Mit welchen antiepileptischen Medikamenten wurde Ihr Hund in der Vergangenheit behandelt und welche Gründe lagen einem Absetzten/Wechsel der Medikamente zugrunde? *

Bitte geben Sie Ihre Antwort hier ein:

Sollte Ihr Hund zuvor noch nicht mit anderen Medikamenten behandelt worden sein, geben sie bitte "Keine" an.

Weiterführende Fragen zur Epilepsie II/III

Wie lange dauert ein durchschnittlicher Krampfanfall ungefähr?

❗ Bitte wählen Sie eine der folgenden Antworten:
Bitte wählen Sie nur eine der folgenden Antworten aus:

- ◯ Wenige Sekunden
- ◯ 1-2 Minuten
- ◯ 3-5 Minuten
- ◯ 6-10 Minuten
- ◯ Länger

Gemeint ist die Zeit, in der der Hund eine motorische Komponente zeigt.

z. B. Zucken, Rudern, rhythmische Kaubewegungen

Hatte Ihr Hund jemals einen Anfall mit einer Dauer von über 5 Minuten, auch als "Status epilepticus" bezeichnet?

❗ Bitte wählen Sie eine der folgenden Antworten:
Bitte wählen Sie nur eine der folgenden Antworten aus:

- ◯ Ja
- ◯ Nein, immer weniger als 5 Minuten.

Hatte Ihr Hund jemals zwei oder mehr Anfälle, ohne dass er Ihnen dazwischen in die Augen schauen konnte?

❗ Bitte wählen Sie eine der folgenden Antworten:
Bitte wählen Sie nur eine der folgenden Antworten aus:

- ◯ Ja
- ◯ Nein, noch nie.

Hatte Ihr Hund jemals mehr als einen Anfall innerhalb von 24 Stunden, auch bekannt als "Cluster-Anfälle"? *

❗ Bitte wählen Sie eine der folgenden Antworten:
Bitte wählen Sie nur eine der folgenden Antworten aus:

◯ Ja, mehr als ein Anfall pro 24 Stunden

◯ Nein, nur ein Anfall pro 24 Stunden

Wie häufig ereignen sich die Krampfanfälle innerhalb von 24 Stunden?

Beantworten Sie diese Frage nur, wenn folgende Bedingungen erfüllt sind:
Antwort war 'Ja, mehr als ein Anfall pro 24 Stunden' bei Frage '30 [ep7]' (Hatte Ihr Hund jemals mehr als einen Anfall innerhalb von 24 Stunden, auch bekannt als "Cluster-Anfälle"?)

❗ Bitte wählen Sie eine der folgenden Antworten:
Bitte wählen Sie nur eine der folgenden Antworten aus:

◯ 2 zeitlich getrennte Anfälle pro 24 Stunden

◯ 3-5 zeitlich getrennte Anfälle pro 24 Stunden

◯ mehr als 5 zeitlich getrennte Anfälle pro 24 Stunden

Weiterführende Fragen zur Epilepsie III/III

Man unterscheidet zwei Arten von Anfällen. Generalisierte Anfälle gehen mit Verkrampfung des gesamten Körpers sowie einem Bewusstseinsverlust einher. Fokale Anfälle entstehen nur in bestimmten Hirngebieten und wirken sich aus diesem Grund nur auf bestimmte Körperregionen aus. Diese können sich als "Ticks", wie plötzlich auftretendes Lecken, Zucken oder Schlucken zeigen. Wie sieht der übliche, durchschnittliche Anfall/Anfallsytp Ihres Hundes aus?

❗ Bitte wählen Sie die zutreffenden Antworten aus:
Bitte wählen Sie alle zutreffenden Antworten aus:

- ☐ Tier fällt auf den Boden und bleibt in Seitenlage
- ☐ Tier ist ansprechbar
- ☐ Tier ist nicht ansprechbar
- ☐ Der Körper erschlafft
- ☐ Der Körper versteift sich
- ☐ Rudernde Bewegungen mit allen 4 Beinen
- ☐ Zucken mit einem Bein
- ☐ Gesichtszucken
- ☐ Kauende Maulbewegungen
- ☐ Rhythmisches Zwinkern
- ☐ Abnormale Augenbewegungen
- ☐ Vergrößerte Pupillen
- ☐ Kopfschütteln
- ☐ Exzessives Speicheln
- ☐ Erbrechen
- ☐ unkontrollierter Harnabsatz
- ☐ unkontrollierter Kotabsatz
- ☐ Krampf beginnt an einer Stelle und breitet sich auf den ganzen Körper aus
- ☐ Verschrecktes und angsterfülltes Verhalten
- ☐ Fliegenschnappen
- ☐ Sonstige: ____________________

Sind die Krampfanfälle auf einen bestimmten Körperteil begrenzt? Bzw. haben Sie jemals fokale Krampfanfälle bei Ihrem Hund beobachtet?

❗ Bitte wählen Sie eine der folgenden Antworten:
Bitte wählen Sie nur eine der folgenden Antworten aus:

- ◯ Nein
- ◯ Ja, auf folgenden Körperteil:

Welche Anzeichen zeigt Ihr Hund üblicherweise während der Erholungsphase nach dem Anfall?

❗ Bitte wählen Sie die zutreffenden Antworten aus:
Bitte wählen Sie alle zutreffenden Antworten aus:

- ☐ Schwankend beim Laufen, "ataktisch"
- ☐ Desorientiert
- ☐ Erstarrt
- ☐ Blind oder sieht schlecht
- ☐ Schläfrig
- ☐ Verängstigt
- ☐ Aggressiv
- ☐ Rastlos, unruhig und wandernd
- ☐ Aktiv am Schnüffeln
- ☐ Hungrig
- ☐ Durstig
- ☐ Anhänglich, möchte mehr Aufmerksamkeit als sonst
- ☐ Normal – keines der oben genannten Anzeichen

Sollte sich Ihr Hund direkt nach einem Anfall vollkommen normal verhalten, wählen sie bitte „Keines der oben genannten Anzeichen“.

Wie lange braucht Ihr Hund im Durchschnitt, um sich von einem üblichen Krampfanfall zu erholen?

❗ Bitte wählen Sie eine der folgenden Antworten:
Bitte wählen Sie nur eine der folgenden Antworten aus:

- ◯ Einige Minuten
- ◯ Bis zu 6 Stunden
- ◯ Bis zu 24 Stunden
- ◯ Bis zu 48 Stunden
- ◯ Mehr als 48 Stunden

Wann hatte Ihr Hund seinen letzten Krampfanfall? *

❗ Bitte wählen Sie eine der folgenden Antworten:
Bitte wählen Sie nur eine der folgenden Antworten aus:

- ◯ Innerhalb der letzten 24 Stunden
- ◯ Innerhalb der letzten 7 Tage
- ◯ Vor 8-14 Tagen
- ◯ Vor 15-21 Tagen
- ◯ Vor 22-28 Tagen
- ◯ Vor 1-3 Monaten
- ◯ Vor 4-6 Monaten
- ◯ Vor 7-12 Monaten
- ◯ Vor über 12 Monaten
- ◯ Ich weiß es nicht.

Sind abgesehen von Epilepsie weitere Erkrankungen bei Ihrem Hund bekannt? *

❗ Bitte wählen Sie eine der folgenden Antworten:
Bitte wählen Sie nur eine der folgenden Antworten aus:

- ◯ Ja
- ◯ Nein

Welche zusätzlichen Erkrankungen liegen vor?

Beantworten Sie diese Frage nur, wenn folgende Bedingungen erfüllt sind:
Antwort war 'Ja' bei Frage '37 [ep11]' (Sind abgesehen von Epilepsie weitere Erkrankungen bei Ihrem Hund bekannt?)

Bitte geben Sie Ihre Antwort hier ein:

Erhält Ihr Hund aktuell andere Medikamente außer der antiepileptischen Präparate oder Impfungen/Entwurmungen? *

❗ Bitte wählen Sie eine der folgenden Antworten:
Bitte wählen Sie nur eine der folgenden Antworten aus:

◯ Ja, noch weitere Medikamente

◯ Nein

Bitte schreiben Sie einen Kommentar zu Ihrer Auswahl

Falls ja, geben Sie im Kommentarfeld bitte die Medikamente und Dosierungen an.

Verhaltensabfrage I/II

Bitte bewerten Sie folgende Aussagen zum Verhalten Ihres Hundes.

*

Bitte wählen Sie die zutreffende Antwort für jeden Punkt aus:

	Nie	Manchmal	Oft	Sehr oft
**Ihr Hund hat einen mühsamen Lernprozess, weil er unachtsam ist oder andere Dinge seine Aufmerksamkeit auf sich ziehen.**	○	○	○	○
**Es fällt Ihrem Hund schwer, sich auf Aufgaben oder Spiele zu konzentrieren.**	○	○	○	○
**Ihr Hund verlässt seinen Platz, obwohl er dort bleiben soll.**	○	○	○	○
**Es scheint, als würde Ihr Hund nicht hören, obwohl er offensichtlich weiß, dass ihn jemand anspricht.**	○	○	○	○

*

Bitte wählen Sie die zutreffende Antwort für jeden Punkt aus:

	Trifft nicht zu	Trifft eher nicht zu	Teils-teils	Trifft eher zu	Trifft zu
**Mein Hund zeigt extreme physische Zeichen, wenn er aufgeregt ist (z.B. Sabbern, Hecheln, Nackenhaar aufstellen, Urinieren, Lippen lecken, Augen aufreißen)**	○	○	○	○	○
**Wenn mein Hund sich aufregt, kann es dazu führen, dass er festes repetitives Verhalten zeigt (d.h. eine Handlung wird auf die gleiche Weise wiederholt; z.B. Schwanz jagen oder im Kreis drehen)**	○	○	○	○	○
**Mein Hund beruhigt sich sehr schnell, nachdem er aufgeregt war.**	○	○	○	○	○

	Trifft nicht zu	Trifft eher nicht zu	Teils-teils	Trifft eher zu	Trifft zu
**Ich würde meinen Hund als sehr impulsiv einschätzen (d.h. er hat einen plötzlichen, starken Drang zu handeln; handelt ohne Voraussicht; handelt ohne Beachtung der Folgen seines Handelns)**	○	○	○	○	○
**Mein Hund scheint sich ohne Grund aufzuregen.**	○	○	○	○	○

Verhaltensabfrage II/II

Bitte bewerten Sie folgende Aussagen zum Verhalten Ihres Hundes.

*

Bitte wählen Sie die zutreffende Antwort für jeden Punkt aus:

	Niemals	Einmal im Monat	Einmal in der Woche	Einmal am Tag	Mehrmals am Tag
**Wie oft geht Ihr Hund auf und ab, läuft im Kreis und/oder wandert ohne Richtung und Ziel umher?**	◯	◯	◯	◯	◯
**Wie oft starrt Ihr Hund leer auf Wände oder den Fußboden?**	◯	◯	◯	◯	◯
**Wie oft steckt Ihr Hund hinter Objekten fest und ist nicht dazu in der Lage, sich zu befreien?**	◯	◯	◯	◯	◯
**Wie oft kommt es vor, dass Ihr Hund Familienmitglieder (Menschen oder Tiere) nicht wahrnimmt?**	◯	◯	◯	◯	◯
**Wie oft läuft Ihr Hund gegen Wände oder Türen?**	◯	◯	◯	◯	◯
**Wie oft läuft Ihr Hund davon, während Sie ihn streicheln, oder vermeidet es, gestreichelt zu werden?**	◯	◯	◯	◯	◯

*

Bitte wählen Sie die zutreffende Antwort für jeden Punkt aus:

	Niemals	1-30 % der Zeit	31-60 % der Zeit	61-99 % der Zeit	Immer
**Hat Ihr Hund Probleme damit Futter zu finden, welches auf den Boden gefallen ist?**	◯	◯	◯	◯	◯

*

Bitte wählen Sie die zutreffende Antwort für jeden Punkt aus:

	Viel weniger	Etwas weniger	Gleichbleibend	Etwas mehr	Viel mehr
**Verglichen mit den letzten 6 Monaten: Läuft Ihr Hund auf und ab, in Kreisen und/oder wandert ohne Grund umher?**	○	○	○	○	○
**Verglichen mit den letzten 6 Monaten: Starrt Ihr Hund leer auf Wände oder Fußböden?**	○	○	○	○	○
**Verglichen mit den letzten 6 Monaten: Uriniert oder kotet Ihr Hund in Bereiche, die er vorher sauber gehalten hat?**	○	○	○	○	○
**Verglichen mit den letzten 6 Monaten: Hat Ihr Hund Probleme, heruntergefallenes Futter auf dem Boden zu finden?**	○	○	○	○	○
**Verglichen mit den letzten 6 Monaten: Nimmt Ihr Hund Familienmitglieder nicht mehr wahr?**	○	○	○	○	○
**Verglichen mit den letzten 6 Monaten: Wie aktiv ist Ihr Hund?**	○	○	○	○	○

Verhaltensprofil (C-BARQ 1)

Wie oft macht Ihr Hund Folgendes? *

Bitte wählen Sie die zutreffende Antwort für jeden Punkt aus:

	Niemals	Selten	Manchmal	Gewöhnlich	Immer
**Kommt auf Rufen sofort zurück, wenn er nicht an der Leine ist.**	○	○	○	○	○
**Gehorcht umgehend auf "Sitz".**	○	○	○	○	○
**Gehorcht umgehend auf "Bleib".**	○	○	○	○	○
**Holt oder versucht Stöcke, Bälle oder Ähnliches zu bringen.**	○	○	○	○	○
**Scheint an allem teilzunehmen oder aufmerksam zuzuhören, was ich tue oder sage.**	○	○	○	○	○
**Ist langsam darin, auf ein Verbot oder eine Belehrung zu reagieren.**	○	○	○	○	○
**Ist langsam darin, neue Tricks oder Fähigkeiten zu erlernen.**	○	○	○	○	○
**Wird leicht von interessanten Anblicken, Geräuschen oder Düften abgelenkt.**	○	○	○	○	○

Reagiert Ihr Hund jemals aggressiv in den folgenden Situationen? *

Bitte wählen Sie die zutreffende Antwort für jeden Punkt aus:

	Niemals	Selten	Manchmal	Gewöhnlich	Immer
**Wenn sich ein unbekannter männlicher Erwachsener nähert, während Sie Gassi gehen oder er an der Leine geführt wird.**	○	○	○	○	○
**Wenn sich eine unbekannte weibliche Erwachsene nähert, während Sie Gassi gehen oder er an der Leine geführt wird.**	○	○	○	○	○
**Wenn sich ein unbekanntes Kind nähert, während Sie Gassi gehen oder er an der Leine geführt wird.**	○	○	○	○	○
**Gegenüber unbekannten Personen, die sich Ihrem Hund nähern, während er in Ihrem Auto ist.**	○	○	○	○	○
**Wenn sich zu Hause eine unbekannte Person dem Besitzer oder einem anderen Familienmitglied nähert.**	○	○	○	○	○

	Niemals	Selten	Manchmal	Gewöhnlich	Immer
**Wenn sich auswärts eine unbekannte Person dem Besitzer oder einem anderen Familienmitglied nähert.**	◯	◯	◯	◯	◯
**Wenn der Postbote oder andere Lieferanten sich dem Zuhause nähern.**	◯	◯	◯	◯	◯
**Wenn Fremde am Zuhause vorbeilaufen, während der Hund im Garten ist.**	◯	◯	◯	◯	◯
**Wenn Jogger, Fahrradfahrer oder Rollschuhfahrer das Zuhause passieren, während der Hund im Garten ist.**	◯	◯	◯	◯	◯
**Gegenüber unbekannten Personen, die den Besitzer zu Hause besuchen.**	◯	◯	◯	◯	◯
**Wenn sich ein unbekannter männlicher Hund nähert, während Sie Gassi gehen oder er an der Leine geführt wird.**	◯	◯	◯	◯	◯
**Wenn sich ein unbekannter weiblicher Hund nähert, während Sie Gassi gehen oder er an der Leine geführt wird.**	◯	◯	◯	◯	◯

	Niemals	Selten	Manchmal	Gewöhnlich	Immer
**Gegenüber unbekannten Hunden, die das Zuhause besuchen.**	○	○	○	○	○

Reagiert Ihr Hund jemals mit aggressivem Verhalten auf die folgenden Situationen? *

Bitte wählen Sie die zutreffende Antwort für jeden Punkt aus:

	Niemals	Selten	Manchmal	Gewöhnlich	Immer
**Wenn der Hund durch ein Haushaltsmitglied belehrt oder bestraft wird.**	○	○	○	○	○
**Wenn dem Hund das Spielzeug, der Knochen oder andere Objekte durch ein Haushaltsmitglied weggenommen werden.**	○	○	○	○	○
**Wenn der Hund durch ein Haushaltsmitglied gebadet oder gepflegt wird.**	○	○	○	○	○
**Wenn der Hund sich direkt einem Haushaltsmitglied nähert, während dieses etwas isst.**	○	○	○	○	○
**Wenn dem Hund durch ein Haushaltsmitglied das Essen weggenommen wird.**	○	○	○	○	○
**Wenn der Hund durch ein Haushaltsmitglied angestarrt wird.**	○	○	○	○	○
**Wenn ein Haushaltsmitglied über den Hund hinweg steigt.**	○	○	○	○	○

	Niemals	Selten	Manchmal	Gewöhnlich	Immer
**Wenn ein Haushaltsmitglied dem Hund gestohlenes Futter/Essen oder Gegenstände wieder wegnimmt.**	◯	◯	◯	◯	◯

Verhaltensprofil (C-BARQ 2)

Bitte beurteilen Sie die folgenden Situationen danach, wie wahrscheinlich Ihr Hund mit angespanntem oder angsterfülltem Verhalten darauf reagieren würde. *

Bitte wählen Sie die zutreffende Antwort für jeden Punkt aus:

	Niemals	Selten	Manchmal	Gewöhnlich	Immer
**Wenn sich ein unbekannter Mann Ihrem Zuhause nähert, während Sie nicht anwesend sind.**	○	○	○	○	○
**Wenn sich eine unbekannte Frau Ihrem Zuhause nähert, während Sie nicht anwesend sind.**	○	○	○	○	○
**Wenn sich ein unbekanntes Kind Ihrem Zuhause nähert, während Sie nicht anwesend sind.**	○	○	○	○	○
**Wenn eine unbekannte Person Sie in Ihrem Zuhause besucht.**	○	○	○	○	○
**In Erwiderung auf schlagartigen oder lauten Krach.**	○	○	○	○	○
**Bei starkem Verkehr.**	○	○	○	○	○
**Als Antwort auf merkwürdige oder unbekannte Objekte auf dem oder in der Nähe des Gehweges.**	○	○	○	○	○
**Während eines Gewitters.**	○	○	○	○	○

	Niemals	Selten	Manchmal	Gewöhnlich	Immer
**Wenn Ihr Hund erstmals einer neuen, unbekannten Situation ausgesetzt ist.**	○	○	○	○	○
**Als Erwiderung auf Wind oder winddurchströmte, bewegte Gegenstände.**	○	○	○	○	○

Zeigt Ihr Hund folgende Verhaltensweisen? *

Bitte wählen Sie die zutreffende Antwort für jeden Punkt aus:

	Niemals	Selten	Manchmal	Gewöhnlich	Immer
**Schütteln, Schlottern oder Zittern, wenn er alleine ist oder kurz bevor er alleine gelassen wird.**	○	○	○	○	○
**Übermäßiges Sabbern, wenn er alleine ist oder kurz bevor er alleine gelassen wird.**	○	○	○	○	○
**Unruhe, Aufregung oder Drangwandern, wenn er alleine ist oder kurz bevor er alleine gelassen wird.**	○	○	○	○	○
**Winseln, wenn er alleine ist oder kurz bevor er alleine gelassen wird.**	○	○	○	○	○
**Bellen, wenn er alleine ist oder kurz bevor er alleine gelassen wird.**	○	○	○	○	○
**Heulen, wenn er alleine ist oder kurz bevor er alleine gelassen wird.**	○	○	○	○	○
**Kauen oder Kratzen an Tür, Boden, Fenstern oder Vorhängen, wenn er alleine ist oder kurz bevor er alleine gelassen wird.**	○	○	○	○	○

	Niemals	Selten	Manchmal	Gewöhnlich	Immer
**Appetitverlust, wenn er alleine ist oder kurz bevor er alleine gelassen wird.**	◯	◯	◯	◯	◯

Was beschreibt das Verhalten Ihres Hundes am besten? *

Bitte wählen Sie die zutreffende Antwort für jeden Punkt aus:

	Niemals	Selten	Manchmal	Gewöhnlich	Immer
**Zeigt eine besondere Anhänglichkeit zu einem bestimmten Haushaltsmitglied.**	○	○	○	○	○
**Neigt dazu einem Haushaltsmitglied von Raum zu Raum innerhalb des Hauses zu folgen.**	○	○	○	○	○
**Tendiert dazu in der Nähe oder in Kontakt zu einem Haushaltsmitglied zu sitzen, wenn dieses sich hinsetzt.**	○	○	○	○	○
**Neigt dazu ein Haushaltsmitglied anzustupsen, sich anzuschmiegen oder die Pfote zu geben, wenn dieses sich hinsetzt.**	○	○	○	○	○
**Wird unruhig und aufgeregt, wenn ein Haushaltsmitglied einer anderen Person Zuwendung schenkt.**	○	○	○	○	○
**Wird unruhig und aufgeregt, wenn ein Haushaltsmitglied einer anderen Hund oder Tier Zuwendung zeigt.**	○	○	○	○	○

	Niemals	Selten	Manchmal	Gewöhnlich	Immer
**Reagiert aggressiv gegenüber Katzen, Eichhörnchen oder anderen Tieren, die den Garten betreten.**	◯	◯	◯	◯	◯
**Jagt Katzen, wenn er die Möglichkeit dazu hat.**	◯	◯	◯	◯	◯
**Jagt Vögel , wenn er die Möglichkeit dazu hat.**	◯	◯	◯	◯	◯
**Jagt Eichhörnchen oder andere kleine Tiere, wenn er die Möglichkeit dazu hat.**	◯	◯	◯	◯	◯

Verhaltensprofil (C-BARQ 3)

Antwortet Ihr Hund in den folgenden Situationen auf eine enorm erregte, aufgeregte Weise? *

Bitte wählen Sie die zutreffende Antwort für jeden Punkt aus:

	Niemals	Selten	Manchmal	Gewöhnlich	Immer
**Wenn ein Haushaltsmitglied nach kurzer Abwesenheit zurückkehrt.**	○	○	○	○	○
**Wenn ein Haushaltsmitglied mit dem Hund spielt.**	○	○	○	○	○
**Wenn es an der Tür klingelt.**	○	○	○	○	○
**Direkt bevor er zum Gassi gehen ausgeführt wird.**	○	○	○	○	○
**Kurz bevor man mit dem Hund eine Autofahrt unternimmt.**	○	○	○	○	○
**Wenn Besucher im Zuhause eintreffen.**	○	○	○	○	○

Reagiert Ihr Hund ängstlich oder verunsichert auf die folgenden Situationen? *

Bitte wählen Sie die zutreffende Antwort für jeden Punkt aus:

	Niemals	Selten	Manchmal	Gewöhnlich	Immer
**Wenn der Hund von einem Tierarzt untersucht oder behandelt wird.**	○	○	○	○	○
**Wenn er von einem Haushaltsmitglied die Krallen geschnitten bekommt.**	○	○	○	○	○
**Wenn er von einem Haushaltsmitglied gepflegt oder gebadet wird.**	○	○	○	○	○
**Wenn sich ein ähnlich großer oder größerer unbekannter Hund nähert.**	○	○	○	○	○
**Wenn sich ein unbekannter Hund von kleinerer Größe nähert.**	○	○	○	○	○

Lebensqualität 1

Bitte geben Sie an, welche Nebenwirkungen der Antiepileptika in den letzten 3 Monaten auftraten und wie stark die Ausprägung war. *

Bitte wählen Sie die zutreffende Antwort für jeden Punkt aus:

	Trat nicht auf	Sehr gering	Gering	Mäßig	Schwer	Sehr schwer
**Gesteigerter Appetit/ Gesteigerte Futteraufnahme**	○	○	○	○	○	○
**Gewichtszunahme**	○	○	○	○	○	○
**Gesteigerte Wasseraufnahme**	○	○	○	○	○	○
**Gesteigerter Harnabsatz**	○	○	○	○	○	○
**Vermehrtes Schlafen**	○	○	○	○	○	○
**Koordinationsprobleme beim Laufen/ schwankender Gang**	○	○	○	○	○	○
**Unruhe/ Drangwandern**	○	○	○	○	○	○
**Juckreiz/ Hautrötungen**	○	○	○	○	○	○
**Erbrechen**	○	○	○	○	○	○
**Durchfall**	○	○	○	○	○	○
**Husten**	○	○	○	○	○	○

*

Bitte wählen Sie die zutreffende Antwort für jeden Punkt aus:

	Stimmt	Stimmt überwiegend	Stimmt teilweise	Stimmt weniger	Stimmt nicht
**Die oben genannten Nebenwirkungen bewerten Sie insgesamt als schwerwiegend.**	○	○	○	○	○
**Die Nebenwirkungen der Antiepileptika waren akzeptabel.**	○	○	○	○	○

Bitte geben Sie an, wie oft die Pflege Ihres Hundes mit idiopathischer Epilepsie in den letzten 3 Monaten Ihr eigenes Leben eingeschränkt hat. *

Bitte wählen Sie die zutreffende Antwort für jeden Punkt aus:

	Nie	Selten	Manchmal	Oft	Sehr oft
**Wie oft hatten Sie das Gefühl, dass die Epilepsie Ihres Hundes im Konflikt mit Ihrer Arbeit / Ausbildung oder Ihren täglichen Aktivitäten steht?**	○	○	○	○	○
**Wie oft hatten Sie das Gefühl, dass die Epilepsie Ihres Hundes Ihr soziales Leben einschränkt?**	○	○	○	○	○
**Wie oft hatten Sie das Gefühl, dass die Epilepsie Ihres Hundes Ihre Unabhängigkeit einschränkt?**	○	○	○	○	○

Lebensqualität 2

Bitte bewerten Sie als Besitzer Ihre Angst über das Auftreten eines Anfalls und über die Auswirkungen auf Ihren Hund in den letzten 3 Monaten. *

Bitte wählen Sie die zutreffende Antwort für jeden Punkt aus:

	Überhaupt nicht	Leicht	Mäßig	Stark	Sehr stark
**Wie sehr waren Sie über die Häufigkeit der Anfälle besorgt?**	○	○	○	○	○
**Wie sehr waren Sie über die Schwere der Anfälle besorgt?**	○	○	○	○	○

Wie sehr haben Sie folgende Nebenwirkungen der Antiepileptika in den vergangenen 3 Monaten gestört? *

Bitte wählen Sie die zutreffende Antwort für jeden Punkt aus:

	Überhaupt nicht	Leicht	Mäßig	Stark	Sehr stark
**Gesteigerter Appetit/ Gesteigerte Futteraufnahme**	○	○	○	○	○
**Gewichtszunahme**	○	○	○	○	○
**Gesteigerte Wasseraufnahme**	○	○	○	○	○
**Gesteigerter Harnabsatz**	○	○	○	○	○
**Vermehrtes Schlafen**	○	○	○	○	○
**Koordinationsprobleme beim Laufen/ schwankender Gang**	○	○	○	○	○
**Unruhe/ Drangwandern**	○	○	○	○	○
**Husten**	○	○	○	○	○

Bitte bewerten Sie Ihre Abneigung gegenüber den Nebenwirkungen der Antiepileptika auf Ihren Hund. *

Bitte wählen Sie die zutreffende Antwort für jeden Punkt aus:

	Überhaupt nicht störend	Geringgradig störend	Störend	Besonders störend	Extrem störend
**Bewerten Sie die körperlichen Auswirkungen der Antiepileptika auf Ihren Hund.**	◯	◯	◯	◯	◯
**Bewerten Sie die geistigen Auswirkungen der Antiepileptika auf Ihren Hund.**	◯	◯	◯	◯	◯

Wie schwerwiegend waren die Enschränkungen, die sich aus der Pflege Ihres Hundes mit idiopathischer Epilepsie für Sie ergeben haben? *

Bitte wählen Sie die zutreffende Antwort für jeden Punkt aus:

	Überhaupt nicht störend	Geringgradig störend	Störend	Besonders störend	Extrem störend
**Bewerten Sie die Einschränkungen bei Ihrer Arbeit / Ausbildung oder Ihren täglichen Aktivitäten.**	○	○	○	○	○
**Bewerten Sie Ihre sozialen Einschränkungen.**	○	○	○	○	○
**Bewerten Sie insgesamt die Einschränkungen, die sich aus der intensiven Pflege Ihres Hunde ergeben.**	○	○	○	○	○

Vielen Dank für Ihre Teilnahme an der Umfrage! Die Ergebnisse der Studie werden Ihnen selbstverständlich mitgeteilt.

Übermittlung Ihres ausgefüllten Fragebogens:
Vielen Dank für die Beantwortung des Fragebogens.
