# Supplementary material for: A six-month prospective, randomised, double-blinded, placebo-controlled, crossover, dietary trial design to investigate the potential of psychobiotics on seizure semiology and comorbidities in canine epilepsy: study protocol
Source: BMC Vet Res. 2023 Mar 3;19:57. doi: 10.1186/s12917-023-03609-0 (PMC9983181; doi:10.1186/s12917-023-03609-0)
Supplement: Supplementary file 2 — Additional file 2. Owner information sheet. Providing detailed information about the aim and procedure of the intervention study. [file 12917_2023_3609_MOESM2_ESM.pdf]

Informationsblatt für Patientenbesitzer zum Studienablauf

„Untersuchung des kurativen Potentials von Psychobiotika auf die Semiologie und Komorbiditäten der kaninen idiopathischen Epilepsie“

Hintergrund:

Epilepsie ist die weltweit am häufigsten auftretende chronische neurologische Erkrankung bei Mensch und Tier. Leider kann einem Drittel der Patienten mit den derzeit zur Verfügung stehenden therapeutischen Möglichkeiten nicht ausreichend geholfen werden. Aus bislang unbekannter Ursache bleibt eine Behandlung mit Antiepileptika bei ihnen erfolglos (therapieresistente Epilepsie). Die daraus resultierende schlechte Anfallskontrolle hat einen negativen Einfluss auf Lebensqualität und Lebenserwartung vieler Patienten und belastet ihre Besitzer ebenfalls enorm. Hinzukommt, dass es sich bei Epilepsie um eine Gehirnerkrankung handelt, die aus mehr als nur den Anfällen besteht. In der gesamten Zeit zwischen den Anfällen leiden ca. 70% der Hunde zusätzlich an Verhaltensänderungen und kognitiven Defiziten, die ihnen das Leben jeden Tag aufs Neue erschweren und derzeit nicht mit in den Behandlungsplan einbezogen werden (z.B. Depressionen, Angstzustände, Trennungsängste und dadurch bedingte übermäßige Anhänglichkeit).

Probiotika-Therapie:

Es gibt stets mehr Hinweise darauf, dass ein gesunder Darm entscheidend für die Gesundheit des Gehirns ist. Beide stehen miteinander in einer engen Verbindung und beeinflussen sich gegenseitig über die sogenannte „Mikrobiom-Darm-Gehirn-Achse“. Dabei könnte die Darmflora (Mikrobiom) einen zentralen Stellenwert bei der Epilepsie-Erkrankung einnehmen, da sie einen maßgeblichen Einfluss auf die Verstoffwechselung des Futters und auch der Antiepileptika haben kann und somit an der Entwicklung von Therapieresistenz beteiligt sein könnte. Eine stabile Darmflora hält den Darm und das Gehirn gesund und beugt Erkrankungen vor. Die Gabe des speziellen Probiotikums dieser Studie fördert nachweislich die Gehirngesundheit und bietet somit einen neuen therapeutischen Ansatz zur Verbesserung der Anfallskontrolle bei Patienten, die nicht auf Antiepileptika ansprechen und lindert zusätzlich ihre Begleiterkrankungen.

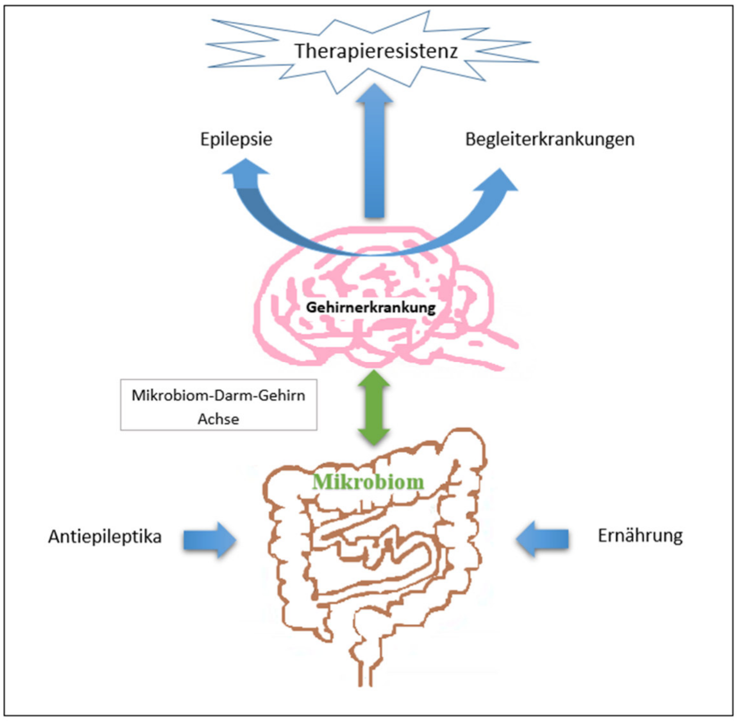

Studienbeginn

Zu Beginn der Studie vereinbaren wir gemeinsam den ersten ambulanten Termin und senden Ihnen die Einverständniserklärung für die Studie zu. Bitte bringen Sie zur **ersten Vorstellung** die **unterzeichnete Einverständniserklärung** und die **Anfallsdokumentation der letzten 6 Monate** (falls vorhanden), sowie die **vorherigen Befunde** der Epilepsie-Abklärung (falls vorhanden) und eine frische **Harnprobe** Ihres Hundes (ca. 3- 5ml) mit. Außerdem beachten Sie bitte, dass Ihr **Hund nüchtern** vorgestellt werden sollte. Beim ersten Klinikbesuch werden wir von Ihnen Informationen zur **Vorgeschichte der Erkrankung** einholen und Ihren Hund umfassend **allgemein und neurologisch untersuchen**. Außerdem finden in Ihrem Beisein drei **Verhaltenstests** statt, in denen die Reaktion Ihres Hundes auf Alltagssituationen beurteilt wird (Dauer der Tests insg. ca. 30 Minuten). Während dieser Zeit wird die Herzfrequenz mit einem Brustgurt erfasst und zusätzlich werden **2x Blut- und 2x Speichelproben** entnommen. Beim Ihrem ersten Klinikbesuch vergeht zwischen den 2 Blutprobenentnahmen **einmalig eine Wartezeit von zwei Stunden**. Diese Zeit können Sie zum Ausfüllen unseres elektronischen **Fragebogens** zum Status quo (allg. Informationen, Verhalten, Epilepsie-Symptomatik und Lebensqualität) nutzen. Sobald alle durchgeführten Untersuchungen und Proben analysiert sind, wird endgültig entschieden, ob Ihr Hund an der Studie teilnehmen kann. Sie erhalten dann entweder das mit dem Probiotikum angereicherte Ergänzungsfuttermittel oder ein Placebo-Präparat über einen Zeitraum von drei Monaten. Danach wechselt Ihr Hund für weitere drei Monate in die jeweils andere Gruppe. Dabei ist weder für Sie noch für den/die untersuchende/n Tierarzt/Tierärztin über den gesamten Verlauf der Studie ersichtlich, um welches Präparat es sich handelt. Solche sogenannten „doppelt-verblindeten" Studien mit Placebo-Gruppen sind besonders aussagekräftig und bilden den Schlüssel für eine gute und sinnvoll durchgeführte Forschungsarbeit, von der Patienten und die Wissenschaft künftig bestmöglich profitieren können. **Falls Ihr Hund bereits mit Antiepileptika behandelt wird, soll diese Therapie während der Studie nicht unterbrochen werden und kann auch, wenn erforderlich, nach tierärztlicher Indikation in Rücksprache angepasst werden!**

Nun sind Sie gefragt!

Im Verlauf der Studie sind **weitere 2 Klinikbesuche und alternierend dazu 2 Telefonate** zur Bewertung des Studienverlaufs nötig. Sie erhalten zu Beginn der Studie einen Terminkalender, in dem bereits alle Klinikbesuche und Telefonate eingetragen sind. Die Einhaltung der vorgegebenen Untersuchungszeitpunkte ist für den Erfolg dieser Studie zwingend erforderlich. Außerdem erhalten Sie eine Fütterungsanleitung und ein tabellarisches Epilepsie-Tagebuch zur Dokumentation der Anfälle, der Fütterung und der Medikation, welches Sie bitte über den gesamten Zeitraum der Studie gewissenhaft ausfüllen.

Telefonate (**2x, im Abstand von 3 Monaten**): Wir rufen Sie an, um uns über den Allgemeinzustand Ihres Hundes, evtl. auftretende Probleme bei der Verabreichung des Probiotikums oder beim Ausfüllen des Epilepsie-Tagebuchs und mögliche Nebenwirkungen zu erkundigen.

Klinikbesuche (**2x, im Abstand von 3 Monaten**): **Bitte bringen Sie zu jedem Besuch das Epilepsietagebuch,** eine **gekühlte frische Kot- und Morgenurinprobe (Auffangen der ersten Harnabsätze)** Ihres Hundes in einer Ihnen zur Verfügung gestellten Styroporbox mit. Zur Datensicherung erstellen wir eine Kopie des Epilepsietagebuches. Während Ihres Besuchs wird Ihnen ein elektronischer **Fragebogen** zum jeweiligen Status quo (allg. Informationen, Verhalten, Epilepsie-Symptomatik und Lebensqualität) Ihres Hundes gegeben, welche Sie bitte bis zum Ende Ihres Aufenthaltes ausfüllen. Ähnlich wie am Tag der ersten Vorstellung werden wir den Studienverlauf auf

Basis Ihrer Informationen protokollieren und Ihren Hund **allgemein und neurologisch untersuchen.** Es werden **1x Blut-** und **2x Speichelproben** entnommen. Außerdem wiederholen wir bei jedem Besuch unsere drei **Verhaltenstests** und messen währenddessen die Herzfrequenz.

Wichtig: Ihr Hund muss vor jedem Klinikbesuch nüchtern sein!
(Das **Ergänzungsfuttermittel** soll unabhängig davon **zur gewohnten Uhrzeit** verabreicht werden, wenn nötig ohne das herkömmliche Hundefutter)

Leere Verpackungen der Ergänzungsfuttermittels bringen Sie bitte zum jeweils nächsten Klinikbesuch zurück. Zum letzten Besuch bringen Sie bitte auch die angebrochenen oder noch nicht geöffneten Verpackungen mit in die Klinik.

Das Ende der Studie

Nach dem letzten Klinikbesuch gilt die Studie als beendet. Die Daten werden zusammengetragen, analysiert und gruppenweise verglichen. Gerne werden wir Sie über das Ergebnis der Studie informieren!

Die Studie wird nach Rücksprache abgebrochen, falls es zu einer starken Verschlechterung der klinischen Epilepsiesymptomatik kommt und diese mit einer Anpassung des Antiepileptika-Profils nicht rückgängig gemacht werden kann. Bei Auftreten von epilepsie-unabhängigen Erkrankungen und einer notwendigen Behandlung kann es ebenfalls nach Rücksprache zum Abbruch der Studie kommen. Weitere Gründe für einen Abbruch sind unwahrscheinlich auftretende gastro-intestinale Nebenwirkungen, die mit einer angemessenen Lebensqualität nicht vereinbar sind. **Sollte sich der Zustand Ihres Hundes verschlechtern bzw. einer der oben genannten Fälle eintreten, so informieren Sie uns bitte umgehend**. Es ist jederzeit ein außerordentlicher Klinikbesuch im Rahmen dieser Studie möglich, bei dem die vorliegende Situation bewertet und über den weiteren Verlauf der Studie entschieden wird. Ein weiterer Grund für den Abbruch der Studie ist die Nichteinhaltung der Versuchsbedingungen durch Sie selbst. Das aktive Austreten aus der Studie ist für Sie -sofern Sie uns darüber informieren- jederzeit möglich. Nur in beiden letzteren Fällen müssen, die seit der ersten Vorstellung angefallenen Kosten von Ihnen getragen werden.
