# Supplementary material for: A six-month prospective, randomised, double-blinded, placebo-controlled, crossover, dietary trial design to investigate the potential of psychobiotics on seizure semiology and comorbidities in canine epilepsy: study protocol
Source: BMC Vet Res. 2023 Mar 3;19:57. doi: 10.1186/s12917-023-03609-0 (PMC9983181; doi:10.1186/s12917-023-03609-0)
Supplement: Supplementary file 3 — Additional file 3. Standardised seizure diary. Owner recordings of seizure frequency, severity, subtype, seizure triggers, antiseizure drug administration, probiotic administration, rescue therapy, adverse events and additional medication. [file 12917_2023_3609_MOESM3_ESM.pdf]

Epilepsie-Tagebuch

Studien-Nr.:	TIHO-EPI-20-01			
Probanden-Nr.:				
Kontrolltermin 0:				
1.Tag Probiotikum:				
	**Tag**	**Angestrebtes Datum**	**Min (-2 Tage)**	**Max (+2 Tage)**
Telefonat 1:	42			
Kontrolltermin 1:	84			
Probiotikum Pause:	85-105			
Probiotikum Beginn:	106			
Telefonat 2:	147			
Kontrolltermin 2:	189			

Klinik für Kleintiere

Tierärztliche Hochschule Hannover, Bünteweg 9, 30559 Hannover

Tel: +49 511 953-6200 Fax: +49 511 953-6203 Email: Teresa.Schmidt@tiho-hannover.de

Studien-Nr.:	TIHO-EPI-20-01
Teilnehmer-Nr.:	
**Form 10: Epilepsie-Tagebuch**	

Hinweise zum Ausfüllen des Formulars

Alle aufgezeichneten Daten müssen Originaldaten sein.
Alle Bereiche müssen vollständig ausgefüllt oder durchgestrichen werden, falls sie nicht verwendet werden.
Alle Formulare müssen leserlich mit dokumentechter und kopierfähiger Tinte ausgefüllt werden.
Alle Formulare müssen unterschrieben und datiert werden.
Fehler dürfen nur so durchgestrichen werden, dass der Originaltext noch lesbar bleibt.
Kein Tipp-Ex® o.Ä. verwenden. Der korrigierte Eintrag muss neben den durchgestrichenen Originaleintrag geschrieben und mit Initialen, Datum und Erklärung (Fehlerkorrektur-Code) versehen werden.

Beispiele für den Fehlerkorrektur-Code finden Sie im nächsten Abschnitt.

Fehlerkorrektur-Code*

EF= Eingabefehler
RF= Rundungsfehler
ÜF= Übertragungsfehler
FP= falsche Probe
UW= unangemessenes Wort
VE= verspäteter Eintrag

RF= Rechtschreibfehler
FK= fehlerhafter Kommentar
FC= falscher Fehlerkorrektur-Code
VÜ= versehentliches Überschreiben

*Bitte kreisen Sie den Fehlerkorrektur-Code ein, um ihn von den Initialen und Datum abzugrenzen.
Die Uhrzeit bitte im 24-Stunden-Format angeben (00:01-24:00).
Datum bitte in TT-MMM-JJJJ angeben (z.B. 12 NOV 2020).
Bitte immer die verwendeten Einheiten angeben. Alle Umrechnungen bitte dokumentieren.

Die ausgefüllten Formulare stellen die benötigten Rohdaten der Studie dar. Zusätzliche Beobachtungen sollen nicht in freie Spalten der Formulare eingetragen werden, sondern separat auf vorgefertigten "Notizzetteln" dokumentiert werden.

Klinik für Kleintiere
Tierärztliche Hochschule Hannover, Bünteweg 9, 30559 Hannover
Tel: +49 511 953-6200 Fax: +49 511 953-6203 Email: Teresa.Schmidt@tiho-hannover.de

Studien-Nr.: TIHO-EPI-20-01
Teilnehmer-Nr.:

Form 10: Epilepsie-Tagebuch

Tag	Datum	Anfälle ?		DZP?*		LEV?*			Anfalls-anzahl	Probiotikum gegeben?	Medikamente gegeben?	Unterschrift	Kommentare
		J	N	J	N	J	N	T					
1													
2													
3													
4													
5													
6													
7													
8													
9													
10													
11													
12													
13													
14													
15													
16													
17													
18													
19													
20													
21													
22													
23													
24													

*Die Anwendung von Diazepam (DZP) kann sowohl bei einer verlängerten Anfallsaktivität (>5 Min.) als auch bei Auftreten von mehr als 3 Anfällen pro Tag angezeigt sein.

*Mit der Levetiracetam-Impulstherapie (LEV) sollte direkt nach dem Auftreten **eines** Anfalls begonnen werden, um Serienanfälle zu vermeiden. Bitte beachten Sie das beigefügte Schema und notieren Sie wie folgt: J=ja, N=nein, T=Tag der Impulstherapie

Unterschrift des Untersuchers: ________________________ Datum (TTMMMJJJJ): ______________________________

Aufgezeichnet von: __________________________________ Datum (TTMMMJJJJ): ______________________________

Studien-Nr.:	TIHO-EPI-20-01
Teilnehmer-Nr.:	

Form 10: Epilepsie-Tagebuch

Tag	Datum	Anfälle ?		DZP?*		LEV?*			Anfalls-anzahl	Probiotikum gegeben?	Medikamente gegeben?	Unterschrift	Kommentare
		J	N	J	N	J	N	T					
25													
26													
27													
28													
29													
30													
31													
32													
33													
34													
35													
36													
37													
38													
39													
40													
41													
42													
43													
44													
45													
46													
47													
48													

*Die Anwendung von Diazepam (DZP) kann sowohl bei einer verlängerten Anfallsaktivität (>5 Min.) als auch bei Auftreten von mehr als 3 Anfällen pro Tag angezeigt sein.

*Mit der Levetiracetam-Impulstherapie (LEV) sollte direkt nach dem Auftreten **eines** Anfalls begonnen werden, um Serienanfälle zu vermeiden. Bitte beachten Sie das beigefügte Schema und notieren Sie wie folgt: J=ja, N=nein, T=Tag der Impulstherapie

Unterschrift des Untersuchers: ________________________ Datum (TTMMMJJJJ): ______________________________

Aufgezeichnet von: __________________________________ Datum (TTMMMJJJJ): ______________________________

Studien-Nr.: TIHO-EPI-20-01
Teilnehmer-Nr.:

Form 10: Epilepsie-Tagebuch

Tag	Datum	Anfälle ?		DZP?*		LEV?*			Anfalls-anzahl	Probiotikum gegeben?	Medikamente gegeben?	Unterschrift	Kommentare
		J	N	J	N	J	N	T					
49													
50													
51													
52													
53													
54													
55													
56													
57													
58													
59													
60													
61													
62													
63													
64													
65													
66													
67													
68													
69													
70													
71													
72													

*Die Anwendung von Diazepam (DZP) kann sowohl bei einer verlängerten Anfallsaktivität (>5 Min.) als auch bei Auftreten von mehr als 3 Anfällen pro Tag angezeigt sein.

*Mit der Levetiracetam-Impulstherapie (LEV) sollte direkt nach dem Auftreten **eines** Anfalls begonnen werden, um Serienanfälle zu vermeiden. Bitte beachten Sie das beigefügte Schema und notieren Sie wie folgt: J=ja, N=nein, T=Tag der Impulstherapie

Unterschrift des Untersuchers: ______________________ Datum (TTMMMJJJJ): ____________________________

Aufgezeichnet von: _______________________________ Datum (TTMMMJJJJ): ___________________________

Studien-Nr.:	TIHO-EPI-20-01
Teilnehmer-Nr.:	

Form 10: Epilepsie-Tagebuch

Tag	Datum	Anfälle ?		DZP?*		LEV?*			Anfalls-anzahl	Probiotikum gegeben?	Medikamente gegeben?	Unterschrift	Kommentare
		J	N	J	N	J	N	T					
73													
74													
75													
76													
77													
78													
79													
80													
81													
82													
83													
84													
85													
86													
87													
88													
89													
90													
91													
92													
93													
94													
95													
96													

*Die Anwendung von Diazepam (DZP) kann sowohl bei einer verlängerten Anfallsaktivität (>5 Min.) als auch bei Auftreten von mehr als 3 Anfällen pro Tag angezeigt sein.

*Mit der Levetiracetam-Impulstherapie (LEV) sollte direkt nach dem Auftreten **eines** Anfalls begonnen werden, um Serienanfälle zu vermeiden. Bitte beachten Sie das beigefügte Schema und notieren Sie wie folgt: J=ja, N=nein, T=Tag der Impulstherapie

Unterschrift des Untersuchers: ________________________ Datum (TTMMMJJJJ): ______________________________

Aufgezeichnet von: __________________________________ Datum (TTMMMJJJJ): ______________________________

Studien-Nr.: TIHO-EPI-20-01
Teilnehmer-Nr.:

Form 10: Epilepsie-Tagebuch

Tag	Datum	Anfälle ?		DZP?*		LEV?*			Anfalls-anzahl	Probiotikum gegeben?	Medikamente gegeben?	Unterschrift	Kommentare
		J	N	J	N	J	N	T					
97													
98													
99													
100													
101													
102													
103													
104													
105													
106													
107													
108													
109													
110													
111													
112													
113													
114													
115													
116													
117													
118													
119													
120													

*Die Anwendung von Diazepam (DZP) kann sowohl bei einer verlängerten Anfallsaktivität (>5 Min.) als auch bei Auftreten von mehr als 3 Anfällen pro Tag angezeigt sein.

*Mit der Levetiracetam-Impulstherapie (LEV) sollte direkt nach dem Auftreten **eines** Anfalls begonnen werden, um Serienanfälle zu vermeiden. Bitte beachten Sie das beigefügte Schema und notieren Sie wie folgt: J=ja, N=nein, T=Tag der Impulstherapie

Unterschrift des Untersuchers: ____________________ Datum (TTMMMJJJJ): ____________________

Aufgezeichnet von: ____________________ Datum (TTMMMJJJJ): ____________________

Studien-Nr.: TIHO-EPI-20-01
Teilnehmer-Nr.:

Form 10: Epilepsie-Tagebuch

Tag	Datum	Anfälle ?		DZP?*		LEV?*			Anfalls-anzahl	Probiotikum gegeben?	Medikamente gegeben?	Unterschrift	Kommentare
		J	N	J	N	J	N	T					
**121**													
**122**													
**123**													
**124**													
**125**													
**126**													
**127**													
**128**													
**129**													
**130**													
**131**													
**132**													
**133**													
**134**													
**135**													
**136**													
**137**													
**138**													
**139**													
**140**													
**141**													
**142**													
**143**													
**144**													

*Die Anwendung von Diazepam (DZP) kann sowohl bei einer verlängerten Anfallsaktivität (>5 Min.) als auch bei Auftreten von mehr als 3 Anfällen pro Tag angezeigt sein.

*Mit der Levetiracetam-Impulstherapie (LEV) sollte direkt nach dem Auftreten **eines** Anfalls begonnen werden, um Serienanfälle zu vermeiden. Bitte beachten Sie das beigefügte Schema und notieren Sie wie folgt: J=ja, N=nein, T=Tag der Impulstherapie

Unterschrift des Untersuchers: __________________________ Datum (TTMMMJJJJ): _________________________________

Aufgezeichnet von: _____________________________________ Datum (TTMMMJJJJ): ________________________________

Studien-Nr.: TIHO-EPI-20-01
Teilnehmer-Nr.:

Form 10: Epilepsie-Tagebuch

Tag	Datum	Anfälle ?		DZP?*		LEV?*			Anfalls-anzahl	Probiotikum gegeben?	Medikamente gegeben?	Unterschrift	Kommentare
		J	N	J	N	J	N	T					
**145**													
**146**													
**147**													
**148**													
**149**													
**150**													
**151**													
**152**													
**153**													
**154**													
**155**													
**156**													
**157**													
**158**													
**159**													
**160**													
**161**													
**162**													
**163**													
**164**													
**165**													
**166**													
**167**													
**168**													

*Die Anwendung von Diazepam (DZP) kann sowohl bei einer verlängerten Anfallsaktivität (>5 Min.) als auch bei Auftreten von mehr als 3 Anfällen pro Tag angezeigt sein.

*Mit der Levetiracetam-Impulstherapie (LEV) sollte direkt nach dem Auftreten **eines** Anfalls begonnen werden, um Serienanfälle zu vermeiden. Bitte beachten Sie das beigefügte Schema und notieren Sie wie folgt: J=ja, N=nein, T=Tag der Impulstherapie

Unterschrift des Untersuchers: ________________________ Datum (TTMMMJJJJ): ______________________________

Aufgezeichnet von: __________________________________ Datum (TTMMMJJJJ): _____________________________

Studien-Nr.: TIHO-EPI-20-01
Teilnehmer-Nr.:

Form 10: Epilepsie-Tagebuch

Tag	Datum	Anfälle ?		DZP?*		LEV?*			Anfalls-anzahl	Probiotikum gegeben?	Medikamente gegeben?	Unterschrift	Kommentare
		J	N	J	N	J	N	T					
169													
170													
171													
172													
173													
174													
175													
176													
177													
178													
179													
180													
181													
182													
183													
184													
185													
186													
187													
188													
189													
190													
191													
192													

*Die Anwendung von Diazepam (DZP) kann sowohl bei einer verlängerten Anfallsaktivität (>5 Min.) als auch bei Auftreten von mehr als 3 Anfällen pro Tag angezeigt sein.

*Mit der Levetiracetam-Impulstherapie (LEV) sollte direkt nach dem Auftreten **eines** Anfalls begonnen werden, um Serienanfälle zu vermeiden. Bitte beachten Sie das beigefügte Schema und notieren Sie wie folgt: J=ja, N=nein, T=Tag der Impulstherapie

Unterschrift des Untersuchers: ________________________ Datum (TTMMMJJJJ): ______________________________

Aufgezeichnet von: __________________________________ Datum (TTMMMJJJJ): ______________________________

Studien-Nr.:	TIHO-EPI-20-01
Teilnehmer-Nr.:	

Form 10: Epilepsie-Tagebuch

Tag	Datum	Anfälle ?		DZP?*		LEV?*			Anfalls-anzahl	Probiotikum gegeben?	Medikamente gegeben?	Unterschrift	Kommentare
		J	N	J	N	J	N	T					
193													
194													
195													
196													
197													
198													
199													
200													
201													
202													
203													
204													
205													
206													

*Die Anwendung von Diazepam (DZP) kann sowohl bei einer verlängerten Anfallsaktivität (>5 Min.) als auch bei Auftreten von mehr als 3 Anfällen pro Tag angezeigt sein.

*Mit der Levetiracetam-Impulstherapie (LEV) sollte direkt nach dem Auftreten **eines** Anfalls begonnen werden, um Serienanfälle zu vermeiden. Bitte beachten Sie das beigefügte Schema und notieren Sie wie folgt: J=ja, N=nein, T=Tag der Impulstherapie

Unterschrift des Untersuchers: ______________________ Datum (TTMMMJJJJ): ____________________________

Aufgezeichnet von: ________________________________ Datum (TTMMMJJJJ): ___________________________
