# Supplementary material for: A six-month prospective, randomised, double-blinded, placebo-controlled, crossover, dietary trial design to investigate the potential of psychobiotics on seizure semiology and comorbidities in canine epilepsy: study protocol
Source: BMC Vet Res. 2023 Mar 3;19:57. doi: 10.1186/s12917-023-03609-0 (PMC9983181; doi:10.1186/s12917-023-03609-0)
Supplement: Supplementary file 6 — Additional file 6: Table 1. Study variables: at each on-site visit, data of primary outcome (behaviour), secondary outcome (seizure semiology) as well as further data of the participants are collected. The table provides a detailed overview of categorising the collected data into preselected study variables and premeditated statistical analysis to evaluate the efficacy of the probiotic (Bifidobacterium longum) in comparison to the placebo supplement. In addition, comparison groups are created of the study phase (phase 1 vs. phase 2), study supplement (probiotic vs. placebo), responder rate (seizure frequency reduction of at least 50%), seizure semiology (seizure type: occurrence of generalised and focal seizures; history/occurrence of cluster seizures, history/occurrence of status epilepticus) and other relevant factors of the questionnaire and behavioural test. [file 12917_2023_3609_MOESM6_ESM.pdf]

Table 1 - Study Variables: At each on-site visit, data of primary outcome (behaviour), secondary outcome (seizure semiology) as well as further data of the participants are collected (data gathered only at certain visits will be marked as: visit 0=V0, visit 1=V1, visit 2=V2). To evaluate the efficacy of the probiotic (*Bifidobacterium longum* BL999) in comparison to the placebo supplement the data will be categorised into preselected study variables and statistical analysis will be conducted as presented below. In addition, comparison groups are established from the following variables: study phase (phase 1 vs. Phase 2), study supplement (probiotic vs. placebo), responder rate (seizure frequency reduction of at least 50%) and seizure semiology (seizure type: occurrence of generalised and focal seizures; history/occurrence of cluster seizures, history/occurrence of status epilepticus). Adhered to previous studies based on a similar study design, the following table provides an overview of the planned statistical analysis [55, 56]. It is modified from Berk et al. 2019 [56].

**Variable measuring points**	**Visit 0**	**1. Interim check-up**	**Visit 1**	**2. Interim check-up**	**Visit 2**
**Study day**	0, before start of study	42±2	84±2	147±2	189±2

**Study variable**	**Variable type**		**Variable type**	**Subtype**	**Statistical tests**	
	**Outcome**	**Covariate**			**Non-parametric**	**Parametric**
**Demographics**						
Body weight (kg)	X		kg	continuous	Wilcoxon-signed rank test	Paired t-test
Age (years)		X	years	continuous	Wilcoxon-signed rank test	Paired t-test

Body temperature (°C)		X	°C	continuous	Wilcoxon-signed rank test	Paired t-test
**Examination**						
Clinical	X	X	abnormal/normal	categorical	Spearman C	Chi-Square X^2 test
Neurological: mentation, behaviour, body posture, gait, cranial nerves, postural reactions, spinal reflexes, sensory exam	X	X	abnormal/normal, grading (0=absent,1=decreased, 2=normal, 3=increased, 4=clonus)	categorical	Spearman C	Chi-Square X^2 test
Visual Analogue Scale (owner assessment of ataxia, sedation, sleep, overall quality of life)	X	X	0-100, change in %	continuous/ categorical	Wilcoxon-signed rank test; Spearman C	Paired t-test; Chi-Square X^2 test
**Seizure data**						
Seizure frequency	X		N per months, study phase, change in %	continuous	Wilcoxon-signed rank test	Paired t-test
Seizure days	X		N per months, study phase, change in %	continuous	Wilcoxon-signed rank test	Paired t-test
Seizure types	X		presence/absence	categorical	Spearman C	Chi-Square X^2 test

Cluster seizure days/occurrence	X		N per months, positive/negative, change in %	continuous/ categorical	Wilcoxon-signed rank test; Spearman C; McNemar-test	Paired t-test; Chi-Square X^2 test; McNemar-test
Status epilepticus	X		N per months, positive/negative	continuous/ categorical	Wilcoxon-signed rank test; Spearman C; McNemar-test	Paired t-test; Chi-Square X^2 test; McNemar-test
Inter-seizure interval	X		In days, months, change in %	continuous/ categorical	Wilcoxon-signed rank test; Spearman C	Paired t-test; Chi-Square X^2 test
Responder rate	X		% of seizure reduction, responder vs. non-responder	continuous/ categorical	Wilcoxon-signed rank test; Spearman C	Paired t-test; Chi-Square X^2 test

**Blood analysis**						
Haematology		X	relative, absolute values	continuous	Wilcoxon-signed rank test	Paired t-test
Blood chemistry		X	U/l, mmol/l, µmol/l, g/l	continuous	Wilcoxon-signed rank test	Paired t-test
Canine pancreatic lipase activity		X	µg/l	continuous	Wilcoxon-signed rank test	Paired t-test
Pre- and postprandial (only at V0) bile acid serum concentration	X		µmol/l	continuous	Wilcoxon-signed rank test	Paired t-test
Drug serum concentration		X	µg/ml	continuous	Wilcoxon-signed rank test	Paired t-test

Serotonin serum level	X		ng/ml	continuous	Wilcoxon-signed rank test	Paired t-test

**Other physiological variables**						
Salvia cortisol levels	X		µg/dl	continuous	Wilcoxon-signed rank test	Paired t-test
Urinalysis (only at V0)	X		positive vs. negative; mg/dl; specific gravity	continuous/ categorical	Wilcoxon-signed rank test; Spearman C	Paired t-test; Chi-Square X^2 test
Neurotransmitter concentration in urine (only at V1 + V2): serotonin, histamine, glycine, phenylethylamine, dopamine, epinephrine, norepinephrine, glutamate, γ- aminobutyric acid	X		µmoles/g creatinine; µg/g creatinine; nmoles/g creatinine	continuous	Wilcoxon-signed rank test	Paired t-test
Metabolome	X		relative/absolute metabolite concentration; mmol/l; g/l	continuous	Wilcoxon-signed rank test	Paired t-test
Microbiome (only at V1 + V2)	X		Operational Taxonomic Units (OTUs); physiological/pathological; Dysbiosis Index (DI)	continuous/ categorical	Wilcoxon-signed rank test; Spearman C	Paired t-test; Chi-Square X^2 test

Questionnaire data						
ADHD RS [59]	X		4 items, option-scaled rating (1-4)	continuous/ categorical	N=2, Wilcoxon-signed rank test; N>2, Kruskal-Wallis-Test; Spearman C	N=2, Paired t-test; N>2, ANOVA; Chi-Square X^2 test
DPQ [61]	X		5 items, option-scaled rating (1-5)	continuous/ categorical	N=2, Wilcoxon-signed rank test; N>2, Kruskal-Wallis-Test; Spearman C	N=2, Paired t-test; N>2, ANOVA; Chi-Square X^2 test
CCDR [62]	X		score: 15-75, option-scaled rating (1-5)	continuous	N=2, Wilcoxon-signed rank test; N>2, Kruskal-Wallis-Test; Spearman C	N=2, Paired t-test; N>2, ANOVA; Chi-Square X^2 test
C-BARQ [63]	X		11 groups, option-scaled rating (0-4)	continuous/ categorical	N=2, Wilcoxon-signed rank test; N>2, Kruskal-Wallis-Test; Spearman C	N=2, Paired t-test; N>2, ANOVA; Chi-Square X^2 test
EpiQoL [64]	X		5 groups, option-scaled rating (1-5/0-5)	continuous/ categorical	N=2, Wilcoxon-signed rank test; N>2, Kruskal-Wallis-Test; Spearman C	N=2, Paired t-test; N>2, ANOVA; Chi-Square X^2 test

**Behavioural test**						
Exploratory behaviour	X		distance, trait occurrence, frequency	continuous/ categorical	Wilcoxon-signed rank test; Spearman C	Paired t-test; Chi-Square X^2 test
Stranger-directed behaviour	X		distance, trait occurrence, frequency	continuous/ categorical	Wilcoxon-signed rank test; Spearman C	Paired t-test; Chi-Square X^2 test
Separation anxiety	X		distance, trait occurrence, frequency	continuous/ categorical	Wilcoxon-signed rank test; Spearman C	Paired t-test; Chi-Square X^2 test
Noise-related anxiety	X		distance, trait occurrence, frequency	continuous/ categorical	Wilcoxon-signed rank test; Spearman C	Paired t-test; Chi-Square X^2 test

List of abbreviations:

[°C: degree Celsius; N: value/amount; U: Unit; U/l: Unit per 1; %: per cent; ADHD RS: attention-deficit/hyperactivity disorder rating scale; DPQ: dog Personality Questionnaire; CCDR: canine cognitive dysfunction rating scale; C-BARQ: canine behavioural assessment & research questionnaire; EpiQoL: evaluation of quality of life in dogs with idiopathic epilepsy]

References

55. Law TH, Davies ES, Pan Y, Zanghi B, Want E, Volk HA. A randomised trial of a medium-chain TAG diet as treatment for dogs with idiopathic epilepsy. Br J Nutr. 2015;114(9):1438-47.

56. Berk BA, Packer RMA, Law TH, Wessmann A, Bathen-Nothen A, Jokinen TS, et al. A double-blinded randomised dietary supplement crossover trial design to investigate the short-term influence of medium chain fatty acid (MCT) supplement on canine idiopathic epilepsy: study protocol. BMC veterinary research. 2019;15(1):181.

59. Vas J, Topal J, Pech E, Miklosi A. Measuring attention deficit and activity in dogs: A new application and validation of a human ADHD questionnaire. Applied Animal Behaviour Science. 2006;103(1-2):105-17.

61. Wright HF, Mills DS, Pollux PMJ. Development and validation of a psychometric tool for assessing impulsivity in the domestic dog (Canis familiaris). International Journal of Comparative Psychology. 2011;24(2):210-25.
62. Salvin HE, McGreevy PD, Sachdev PS, Valenzuela MJ. The canine cognitive dysfunction rating scale (CCDR): a data-driven and ecologically relevant assessment tool. Veterinary journal. 2011;188(3):331-6.
63. Hsu Y, Serpell JA. Development and validation of a questionnaire for measuring behavior and temperament traits in pet dogs. J Am Vet Med Assoc. 2003;223(9):1293-300.
64. Wessmann A, Volk HA, Parkin T, Ortega M, Anderson TJ. Evaluation of quality of life in dogs with idiopathic epilepsy. J Vet Intern Med. 2014;28(2):510-4.
